# Supplementary material for: Midwifery continuity of care: A scoping review of where, how, by whom and for whom?
Source: PLOS Glob Public Health. 2022 Oct 5;2(10):e0000935. doi: 10.1371/journal.pgph.0000935 (PMC10021789; doi:10.1371/journal.pgph.0000935)
Supplement: S1 Text — (DOCX) [file pgph.0000935.s001.docx]

**S1_Text: Search strategy**

Midwifery Continuity of Care scoping review of global implementation initiatives systematic search involved the following electronic databases MEDLINE, Embase, CENTRAL, CINAHL, PsychINFO, Web of Science, using search terms and keywords with no limit on language. In addition, we searched PubMed, Google Scholar and Scopus. To identify unpublished or ongoing trials we searched Dimensions, PROSPERO, the WHO International Clinical Trials Registry platform.

**MEDLINE Search Strategy**

1 midwif*.mp.

2 exp Nurse Midwives/

3 nurse.mp. or exp Nurses/

4 obstetric nurse.mp. or exp Obstetric Nursing/

5 exp Physicians, Family/

6 physician.mp. or exp Physicians/

7 exp Obstetrics/ or obstetrician.mp.

8 exp Midwifery/

9 exp Maternal Health Services/ or maternity care.mp.

10 antenatal care.mp. or exp Prenatal Care/

11 Perinatal Care/

12 exp Perinatal Care/ or exp Delivery, Obstetric/ or intrapartum care.mp.

13 postnatal care.mp. or exp Postnatal Care/

14 continuum.mp.

15 continuity of care.mp. or exp "Continuity of Patient Care"/

16 caseload*.mp.

17 known midwife.mp.

18 exp Case Management/

19 exp "Delivery of Health Care"/ and continuity.mp.

20 ((nurs* or midwif*) adj2 (care* or team* or led*)).mp. [mp=title, abstract, original title, name of substance word, subject heading word, floating sub-heading word, keyword heading word, organism supplementary concept word, protocol supplementary concept word, rare disease supplementary concept word, unique identifier, synonyms]

21 1 or 2 or 3 or 4 or 5 or 6 or 7

22 8 or 9 or 10 or 11 or 12 or 13 or 14

23 15 or 16 or 17 or 18 or 19 or 20

24 21 and 22 and 23

25 limit 24 to (humans and yr="2012 -Current")

**EMBASE**

| 1. | exp midwife/ or midwif*.mp. |
| --- | --- |
| 2. | exp nurse/ or exp nurse midwife/ |
| 3. | obstetric nurse.mp. |
| 4. | exp physician/ |
| 5. | exp obstetrician/ |
| 6. | family physician.mp. or exp general practitioner/ |
| 7. | midwifery.mp. |
| 8. | maternity care.mp. or exp maternal care/ |
| 9. | exp perinatal care/ |
| 10. | antenatal care.mp. or exp prenatal care/ |
| 11. | pregnancy care.mp. |
| 12. | exp intrapartum care/ or exp obstetric care/ |
| 13. | (labo?r and birth care).mp. [mp=title, abstract, heading word, drug trade name, original title, device manufacturer, drug manufacturer, device trade name, keyword heading word, floating subheading word, candidate term word] |
| 14. | exp postnatal care/ |
| 15. | postpartum care.mp. |
| 16. | continuum.mp. |
| 17. | continuity.mp. and patient care/ |
| 18. | continuity of care.mp. |
| 19. | caseload*.mp. |
| 20. | known midwife.mp. |
| 21. | exp case management/ |
| 22. | 1 or 2 or 3 or 4 or 5 or 6 |
| 23. | 7 or 8 or 9 or 10 or 11 or 12 or 13 or 14 or 15 or 16 |
| 24. | 17 or 18 or 19 or 20 or 21 |
| 25. | 22 and 23 and 24 |
| 26. | limit 25 to (human and yr="2012 -Current") |

**CENTRAL Search Strategy**

1ID Search Hits

#1 MeSH descriptor: [Midwifery] explode all trees

#2 MeSH descriptor: [Nursing] explode all trees

#3 MeSH descriptor: [Obstetric Nursing] explode all trees

#4 MeSH descriptor: [Nurse Midwives] explode all trees

#5 MeSH descriptor: [General Practitioners] explode all trees

#7 Obstetricians

#8 midwif* OR nurs*

#9 maternity care

#10 antenatal care OR Pregnancy care

#11 MeSH descriptor: [Perinatal Care] explode all trees

#12 perinatal care

#13 Labo?r and birth

#14 postnatal care

#15 midwifery

#16 obstetrics

#17 continuity of care OR continuity OR continuum

#18 caseload* OR case management

#19 known midwife

#20 MeSH descriptor: [Continuity of Patient Care] explode all trees

#21 #1 OR #2 OR #3 OR #4 OR #5 OR #6 OR #7 OR #8

#22 #9 OR #10 OR #11 OR #12 OR #13 OR #14 OR #15 OR #16

#23 #17 OR #18 OR #19 OR #20

#24 #21 AND #22 AND #23 with Cochrane Library publication date Between Jan 2012 and Jan 2022

**CINAHL Search Strategy**

S1 exp midwife or midwives

S2 ep obstetric nurse OR obgyn nurse

S3 exp maternal child health nurse OR ( maternal child and family health nurse )

S4 exp general practitioner or gp or family doctor or physician

S5 exp obstetrician

S6 exp midwifery or midwifery care

S7 exp maternity care' or 'antenatal care' or 'prenatal care' or 'obstetric care

S8 exp postnatal care or postpartum or early postnatal period or care after birth or care following birth

S9 exp intrapartum care OR perinatal care

S10 exp continuum or continuum of care

S11 exp continuity of care OR continuity of patient care OR ( continuity of carer or caseload midwifery or known midwife or midwife led continuity of care )

S12 exp case manag* or caseload*

S13 exp continuity AND ( team or model )

S14 S1 or S2 or S3 or S4 or S5

S15 S6 or S8 or S9

S16 S10 or S11 or S12 or S13

S17 S14 and S15 and S16 Limit publication year: 2012-2022; Human

**PsychINFO Search Strategy**

1 midwif*.mp. [mp=title, abstract, heading word, table of contents, key concepts, original title, tests & measures, mesh word]

2 exp Nurses/

3 exp Nursing/

4 exp General Practitioners/

5 exp Family Physicians/

6 exp Obstetricians/

7 exp Midwifery/

8 exp Prenatal Care/ or exp Antepartum Period/

9 exp Obstetrics/

10 childbirth.mp. or exp Birth/

11 exp Perinatal Period/ or perinatal care.mp.

12 exp Postnatal Period/ or postnatal care.mp.

13 pregnancy care.mp.

14 postpartum care.mp.

15 continuity of care.mp. or exp "Continuum of Care"/

16 continuity.mp.

17 continuum.mp.

18 exp Case Management/ or caseload*.mp.

19 1 or 2 or 3 or 4 or 5 or 6

20 7 or 8 or 9 or 10 or 11 or 12 or 13 or 14

21 15 or 16 or 17 or 18

22 19 and 20 and 21

23 limit 22 to (human and yr="2012 -Current")

**Web of Science**

https://www.webofscience.com/wos/alldb/summary/47fbd2c0-5e35-401c-addd-2ec7851c79df-1f1c2232/relevance/1
