# Supplementary material for: Midwifery continuity of care: A scoping review of where, how, by whom and for whom?
Source: PLOS Glob Public Health. 2022 Oct 5;2(10):e0000935. doi: 10.1371/journal.pgph.0000935 (PMC10021789; doi:10.1371/journal.pgph.0000935)
Supplement: S1 Table — (DOCX) [file pgph.0000935.s002.docx]

**S1 Table: All included items**

**Supplementary file: Midwifery Continuity of Care initiatives**

| **Unique study** | **Item** | **Country** | **Income level** | **Study design** | **Setting** | **Model of care** | **Continuity of care** | **Providers** | **Special populations** | **Focus** | **Community or facility based** | **Type of publication** |
| --- | --- | --- | --- | --- | --- | --- | --- | --- | --- | --- | --- | --- |
| yes | Allen 2015^1^ | Australia | HIC | Quantitative | Urban | Caseload midwifery | Antenatal, intrapartum, postpartum | Midwives | Young/adolescent women | Women's outcomes | Community | Peer reviewed paper |
| yes | Allen 2015^2^ | Australia | HIC | Quantitative | Urban | Caseload midwifery | Antenatal, intrapartum, postpartum | Midwives | Young/adolescent women | Women's experiences | Community | Peer reviewed paper |
| yes | Allen 2016^3^ | Australia | HIC | Quantitative | Urban | Caseload midwifery | Antenatal, intrapartum, postpartum | Midwives | Young/adolescent women | Women's experiences | Community | Peer reviewed paper |
| yes | Anwar 2014^4^ | Pakistan | LMIC | Qualitative study | Urban | Midwife-led continuity | Antenatal, intrapartum, postpartum | Midwives |  | Women's experiences | Hospital based | Peer reviewed paper |
| yes | Aune 2012^5^ | Norway | HIC | Qualitative study | Urban | Midwife-led continuity | Antenatal, intrapartum, postpartum | Midwifery students |  | Midwifery students | Hospital based | Peer reviewed paper |
| yes | Aune 2013^6^ | Norway | HIC | Qualitative study | Urban | Midwife-led continuity | Antenatal, intrapartum, postpartum | Midwives |  | Women's outcomes | Hospital based | Peer reviewed paper |
| yes | Aune 2021^7^ | Norway | HIC | Qualitative study | Urban | Midwife-led continuity | Antenatal, intrapartum, postpartum | Midwives |  | Midwives experiences | Hospital based | Peer reviewed paper |
| yes | Baird 2021^8^ | Australia | HIC | Qualitative study | Urban | Midwifery group practice | Antenatal, intrapartum, postpartum | Midwifery students |  | Midwifery students | Hospital based | Peer reviewed paper |
| yes | Barimani 2012^9^ | Sweden | HIC | Qualitative study | Urban | Continuity of care | Antenatal, intrapartum, postpartum | Midwives and child health nurses |  | Women's experiences | Community | Peer reviewed paper |
| yes | Barker, 2020^10^ | Australia | HIC | Qualitative study | Urban | Midwife-led continuity | Antenatal, intrapartum, postpartum | Midwives |  | Midwives experiences | Hospital based | Conference Abstract |
| yes | Beake 2013^11^ | United Kingdom | HIC | Qualitative study | Urban | Caseload midwifery | Antenatal, intrapartum, postpartum | Midwives |  | Women's experiences | Hospital based | Peer reviewed paper |
| yes | Beckmann 2012^12^ | Australia | HIC | Quantitative | Urban | Midwifery group practice | Antenatal, intrapartum, postpartum | Midwives |  | Women's outcomes | Hospital based | Peer reviewed paper |
| yes | Biswas 2018^13^ | Bangladesh | LMIC | Quantitative | Rural | Midwife-led continuity | Antenatal, intrapartum, postpartum | Midwives | Teagarden workers | Women's outcomes | Community | Peer reviewed paper |
| yes | Brintworth 2013^14^ | United Kingdom | HIC | Qualitative study | Urban | Caseload midwifery | Antenatal, intrapartum, postpartum | Midwives |  | Model of care implementation | Community | Peer reviewed paper |
| yes | Browne 2014^15^ | Australia | HIC | Survey study | Urban | Midwifery group practice | Antenatal, intrapartum, postpartum | Midwifery students |  | Women's experiences | Hospital based | Peer reviewed paper |
| yes | Burau 2015^16^ | Denmark | HIC | Qualitative study | Urban | Caseload midwifery | Antenatal, intrapartum, postpartum | Midwives |  | Model of care implementation | Hospital based | Peer reviewed paper |
| yes | Butcher 2016^17^ | United Kingdom | HIC | Quantitative | Urban | Midwife-led continuity | Antenatal, postpartum | Midwives | Young/adolescent women | Women's outcomes | Hospital based | Peer reviewed paper |
| yes | Butska, 2020^18^ | Canada | HIC | Survey study | Urban | Midwife-led continuity | Antenatal, intrapartum, postpartum | Midwives | Refugee women | Midwives experiences | Community | Conference Abstract |
| yes | Callander 2021^19^ | Australia | HIC | Health economics analysis | Urban | Midwifery group practice | Antenatal, intrapartum, postpartum | Midwives |  | Cost analysis | Hospital based | Peer reviewed paper |
| yes | Carter 2015^20^ | Australia | HIC | Survey study | Rural | Midwifery group practice | Antenatal, intrapartum, postpartum | Midwifery students |  | Midwifery students | Hospital based | Peer reviewed paper |
| yes | Churchill 2020^21^ | Canada | HIC | Qualitative study | Urban | Midwife-led continuity | Antenatal, intrapartum, postpartum | Midwives | Indigenous women | Women's experiences | Community | Peer reviewed paper |
| yes | Collins 2014^22^ | United Kingdom | HIC | Quantitative | Urban | Midwife-led continuity | Antenatal, intrapartum, postpartum | Midwives |  | Women's outcomes | Hospital based | Peer reviewed paper |
| yes | Cummins 2015^23^ | Australia | HIC | Qualitative study | Urban | Midwifery group practice | Antenatal, intrapartum, postpartum | Midwives |  | Midwives experiences | Hospital based | Peer reviewed paper |
| yes | Cummins 2021^24^ | Australia | HIC | Qualitative study | Urban | Midwife-led continuity | Antenatal, postpartum | Midwives |  | Women's experiences | Hospital based | Peer reviewed paper |
| yes | Dahlberg 2016^25^ | Norway | HIC | Qualitative study | Urban | Midwife-led continuity | Antenatal, postpartum | Midwives |  | Women's experiences | Hospital based | Peer reviewed paper |
| yes | Daigle, 2020^26^ | Canada | HIC | Qualitative study | Unknown | Midwife-led continuity | Antenatal, intrapartum, postpartum | Midwives |  | Women's experiences | Community | Conference Abstract |
| yes | Davison 2015^27^ | Australia | HIC | Qualitative study | Urban | Caseload midwifery - private | Antenatal, intrapartum, postpartum | Midwives |  | Women's experiences | Community | Peer reviewed paper |
| yes | Dawson 2016^28^ | Australia | HIC | Survey study | Both | Caseload midwifery | Antenatal, intrapartum, postpartum | Midwives |  | Managers | Hospital based | Peer reviewed paper |
| no | Dawson 2018^29^ | Australia | HIC | Survey study | Both | Caseload midwifery | Antenatal, intrapartum, postpartum | Midwives |  | Managers | Hospital based | Peer reviewed paper |
| yes | deJonge 2014^30^ | Netherlands | HIC | Qualitative study | Urban | Midwife-led continuity | Antenatal, intrapartum, postpartum | Midwives |  | Women's experiences | Community | Peer reviewed paper |
| yes | Denham 2017^31^ | United Kingdom | HIC | Quantitative | Rural | Midwife-led continuity | Antenatal, intrapartum, postpartum | Midwives |  | Women's outcomes | Hospital based | Peer reviewed paper |
| yes | Dery 2020^32^ | Ghana | LMIC | Quantitative | Rural | Continuity of care | Antenatal, intrapartum, postpartum | Midwives or doctors |  | Women's outcomes | Hospital based | Peer reviewed paper |
| yes | deWolff 2021^33^ | Denmark | HIC | Trial | Urban | Midwife-led continuity | Antenatal, intrapartum, postpartum | Midwives | Women with pre-existing conditions | Women's outcomes | Hospital based | Peer reviewed paper |
| yes | Dharni 2021^34^ | United Kingdom | HIC | Qualitative study | Urban | Midwife-led continuity | Antenatal, postpartum | Midwives |  | Women's outcomes | Hospital based | Peer reviewed paper |
| yes | Dixon 2017^35^ | New Zealand | HIC | Survey study | Both | Caseload midwifery | Antenatal, intrapartum, postpartum | Midwives |  | Midwives experiences | Community | Peer reviewed paper |
| yes | Dobbs, 2020^36^ | Australia | HIC | Practice story | Rural | Midwifery group practice | Antenatal, intrapartum, postpartum | Midwives |  | Model of care implementation | Hospital based | Conference Abstract |
| yes | Doering, 2020^37^ | Japan | HIC | Qualitative study | Unknown | Midwife-led continuity | Antenatal, intrapartum, postpartum | Midwives |  | Women's experiences | Birth centre | Conference Abstract |
| yes | Durst 2016^38^ | Australia | HIC | Quantitative | Urban | Midwifery group practice | Antenatal, intrapartum, postpartum | Midwives |  | Women's outcomes | Hospital based | Peer reviewed paper |
| yes | Edmondson 2014^39^ | Australia | HIC | Qualitative study | Rural | Caseload midwifery | Antenatal, intrapartum, postpartum | Midwives |  | Midwives experiences | Hospital based | Peer reviewed paper |
| yes | Fenwick 2017^40^ | Australia | HIC | Qualitative study | Both | Caseload midwifery - private | Antenatal, intrapartum, postpartum | Midwives |  | Women's experiences | Hospital based | Peer reviewed paper |
| yes | Fenwick 2018^41^ | Australia | HIC | Survey study | Urban | Midwifery group practice | Antenatal, intrapartum, postpartum | Midwives |  | Midwives experiences | Hospital based | Peer reviewed paper |
| yes | Fernandez Turienzo 2020^42^ | United Kingdom | HIC | Trial | Urban | Midwifery group practice | Antenatal, intrapartum, postpartum | Midwives, obstetricians | Risk of preterm birth | Women's experiences | Hospital based | Peer reviewed paper |
| yes | Fernandez Turienzo 2020^43^ | United Kingdom | HIC | Trial | Urban | Midwifery group practice | Antenatal, intrapartum, postpartum | Midwives, obstetricians | Risk of preterm birth | Women's outcomes | Hospital based | Peer reviewed paper |
| yes | Flora 2018^44^ | Australia | HIC | Quantitative | Urban | Midwife-led continuity | Antenatal, postpartum | Midwives, social worker | Drug or alcohol dependence | Model of care implementation | Hospital based | Conference Abstract |
| yes | Floris 2018^45^ | Switzerland | HIC | Survey study | Urban | Team midwifery | Antenatal, intrapartum, postpartum | Midwives |  | Women's experiences | Hospital based | Peer reviewed paper |
| yes | Foster 2021^46^ | Australia | HIC | Quantitative | Urban | Caseload midwifery | Antenatal, intrapartum, postpartum | Midwifery students |  | Midwifery students | Hospital based | Peer reviewed paper |
| Yes | Fox 2013^47^ | Singapore | HIC | Quantitative | Urban | Caseload midwifery | Antenatal, intrapartum, postpartum | Midwives |  | Women's outcomes | Hospital based | Peer reviewed paper |
| yes | Fujita, 2018^48^ | Japan | HIC | Survey study | Urban | Midwife-led continuity | Antenatal, intrapartum, postpartum | Midwives |  | Midwives experiences | Birth centre | Peer reviewed paper |
| yes | Gao 2014^49^ | Australia | HIC | Quantitative | Rural | Midwifery group practice | Antenatal, intrapartum, postpartum | Midwives |  | Cost analysis | Hospital based | Peer reviewed paper |
| yes | Gidaszewski 2019^50^ | Australia | HIC | Quantitative | Urban | Caseload midwifery | Antenatal, intrapartum, postpartum | Midwives |  | Women's outcomes | Hospital based | Peer reviewed paper |
| yes | Gilkison 2015^51^ | New Zealand | HIC | Qualitative study | Both | Caseload midwifery | Antenatal, intrapartum, postpartum | Midwives |  | Midwives experiences | Community | Peer reviewed paper |
| yes | Goodman 2015^52^ | USA | HIC | Practice story | Urban | Midwife-led continuity | Antenatal, postpartum | Midwives | Drug or alcohol dependence | Model of care implementation | Hospital based | Peer reviewed paper |
| yes | Gray 2012^53^ | Australia | HIC | Survey study | Both | Caseload midwifery | Antenatal, intrapartum, postpartum | Midwifery students |  | Midwifery students | Hospital based | Peer reviewed paper |
| yes | Gray 2013^54^ | Australia | HIC | Qualitative study | Both | Caseload midwifery | Antenatal, intrapartum, postpartum | Midwifery students |  | Midwifery students | Hospital based | Peer reviewed paper |
| yes | Griffiths 2013^55^ | New Zealand | HIC | Qualitative study | Both | Caseload midwifery | Antenatal, intrapartum, postpartum | Midwives | Socially disadvantaged or vulnerable women | Midwives experiences | Community | Peer reviewed paper |
| yes | Gross, 2018^56^ | Germany | HIC | Survey study | Both | Midwife-led continuity | Antenatal, intrapartum, postpartum | Midwives |  | Midwives experiences | Hospital based | Peer reviewed paper |
| yes | Grylka-Baeschlin 2020^57^ | Switzerland | HIC | Quantitative | Urban | Midwife-led continuity | Antenatal, intrapartum, postpartum | Midwives |  | Women's outcomes | Birth centre | Peer reviewed paper |
| yes | Gu, 2013^58^ | China | LMIC | Trial | Urban | Caseload midwifery | Antenatal, intrapartum | Midwives, obstetricians |  | Women's outcomes | Hospital based | Peer reviewed paper |
| yes | Hadebe 2021^59^ | United Kingdom | HIC | Quantitative | Urban | Caseload midwifery | Antenatal, intrapartum, postpartum | Midwives | Socially disadvantaged or vulnerable women | Women's outcomes | Hospital based | Peer reviewed paper |
| yes | Hailemeskel 2021^60^ | Ethiopia | LMIC | Qualitative study | Rural | Midwife-led continuity | Antenatal, intrapartum, postpartum | Midwives |  | Midwives experiences | Hospital based | Peer reviewed paper |
| yes | Hailemeskel 2021^61^ | Ethiopia | LMIC | Trial | Rural | Midwife-led continuity | Antenatal, intrapartum, postpartum | Midwives |  | Women's outcomes | Hospital based | Peer reviewed paper |
| yes | Haines 2015^62^ | Australia | HIC | Quantitative | Rural | Caseload midwifery | Antenatal, intrapartum, postpartum | Midwives |  | Women's outcomes | Hospital based | Peer reviewed paper |
| yes | Hardeman 2020^63^ | USA | HIC | Quantitative | Urban | Midwife-led continuity | Antenatal, intrapartum, postpartum | Midwives | African American women | Model of care implementation | Birth centre | Peer reviewed paper |
| yes | Hartz 2019^64^ | Australia | HIC | Quantitative | Urban | Midwifery group practice | Antenatal, intrapartum, postpartum | Midwives, Aboriginal Health Worker | Indigenous women | Women's outcomes | Community | Peer reviewed paper |
| yes | Hewitt 2021^65^ | Australia | HIC | Qualitative study | Urban | Midwifery group practice | Antenatal, intrapartum, postpartum | Midwives |  | Midwives experiences | Hospital based | Peer reviewed paper |
| yes | Hildingsson 2018^66^ | Sweden | HIC | Survey study | Urban | Caseload midwifery | Antenatal, intrapartum, postpartum | Midwives |  | Women's experiences | Hospital based | Peer reviewed paper |
| yes | Hildingsson 2018^66^ | Sweden | HIC | Survey study | Urban | Caseload midwifery | Antenatal, intrapartum | Midwives |  | Women's experiences | Hospital based | Peer reviewed paper |
| no | Hildingsson 2019^67^ | Sweden | HIC | Survey study | Urban | Caseload midwifery | Antenatal, intrapartum | Midwives |  | Women's experiences | Hospital based | Peer reviewed paper |
| yes | Hildingsson 2020^68^ | Sweden | HIC | Quantitative | Urban | Caseload midwifery | Antenatal, intrapartum, postpartum | Midwives |  | Women's outcomes | Hospital based | Peer reviewed paper |
| yes | Hildingsson 2020^69^ | Sweden | HIC | Survey study | Rural | Caseload midwifery | Antenatal, intrapartum, postpartum | Midwives |  | Women's experiences | Hospital based | Peer reviewed paper |
| no | Hildingsson 2021^70^ | Sweden | HIC | Survey study | Urban | Caseload midwifery | Antenatal, intrapartum, postpartum | Midwives |  | Women's experiences | Hospital based | Peer reviewed paper |
| yes | Hildingsson 2021^71^ | Sweden | HIC | Quantitative | Rural | Midwife-led continuity | Antenatal, intrapartum | Midwives |  | Women's experiences | Hospital based | Peer reviewed paper |
| yes | Hollins Martin 2020^72^ | United Kingdom | HIC | Survey study | Urban | Midwife-led continuity | Antenatal, intrapartum, postpartum | Midwives |  | Midwives experiences | Hospital based | Peer reviewed paper |
| yes | Holroyd, 2019^73^ | Sweden | HIC | Qualitative study | Urban | Midwifery group practice | Antenatal, intrapartum | Midwives |  | Midwives experiences | Hospital based | Published report |
| yes | Homer 2012^74^ | Australia | HIC | Quantitative | Urban | Midwifery group practice | Antenatal, intrapartum, postpartum | Midwives, Aboriginal Health Worker | Indigenous women | Women's outcomes | Community | Peer reviewed paper |
| yes | Homer 2017^75^ | United Kingdom | HIC | Quantitative | Urban | Midwifery group practice | Antenatal, intrapartum, postpartum | Midwives | Socially disadvantaged or vulnerable women | Women's outcomes | Community | Peer reviewed paper |
| yes | Homer 2021^76^ | Australia | HIC | Trial | Urban | Midwifery group practice | Antenatal, intrapartum, postpartum | Midwives | Previous CS | Women's outcomes | Hospital based | Peer reviewed paper |
| yes | Hopkinson, 2020^77^ | Australia | HIC | Qualitative study | Urban | Caseload midwifery | Antenatal, intrapartum, postpartum | Midwives |  | Midwives experiences | Hospital based | Conference Abstract |
| yes | Hua 2018^78^ | China | LMIC | Quantitative | Urban | Midwife-led continuity | Antenatal, intrapartum, postpartum | Midwives |  | Women's outcomes | Hospital based | Peer reviewed paper |
| no | Hunter 2017^79^ | New Zealand | HIC | Qualitative study | Both | Caseload midwifery | Antenatal, intrapartum, postpartum | Midwives |  | Midwives experiences | Community | Peer reviewed paper |
| yes | Iida 2012^80^ | Japan | HIC | Survey study | Urban | Midwife-led continuity | Antenatal, intrapartum, postpartum | Midwives |  | Women's experiences | Birth centre | Peer reviewed paper |
| yes | Iida 2014^81^ | Japan | HIC | Survey study | Urban | Midwife-led continuity | Antenatal, intrapartum, postpartum | Midwives |  | Women's experiences | Birth centre | Peer reviewed paper |
| yes | Iida 2021^82^ | Japan | HIC | Qualitative study | Urban | Team midwifery | Antenatal, intrapartum, postpartum | Midwives |  | Women's experiences | Birth centre | Peer reviewed paper |
| yes | Jepsen 2015^83^ | Denmark | HIC | Qualitative study | Urban | Caseload midwifery | Antenatal, intrapartum, postpartum | Midwives |  | Midwives experiences | Hospital based | Peer reviewed paper |
| yes | Jepsen 2017^84^ | Denmark | HIC | Survey study | Urban | Caseload midwifery | Antenatal, intrapartum, postpartum | Midwives |  | Midwives experiences | Hospital based | Peer reviewed paper |
| yes | Jepsen 2018^85^ | Denmark | HIC | Quantitative | Urban | Caseload midwifery | Antenatal, intrapartum, postpartum | Midwives |  | Women's outcomes | Hospital based | Peer reviewed paper |
| yes | Josif 2014^86^ | Australia | HIC | Qualitative study | Rural | Midwifery group practice | Antenatal, intrapartum, postpartum | Midwives, Aboriginal Health Worker | Indigenous women | Women's experiences | Community | Peer reviewed paper |
| yes | Kashani 2021^87^ | Australia | HIC | Qualitative study | Rural | Midwifery group practice | Antenatal, intrapartum, postpartum | Midwives |  | Midwives experiences | Hospital based | Peer reviewed paper |
| yes | Kelly 2014^88^ | Australia | HIC | Qualitative study | Both | Caseload midwifery | Antenatal, intrapartum, postpartum | Midwifery students | Indigenous women | Women's experiences | Hospital based | Peer reviewed paper |
| yes | Kenny 2015^89^ | Ireland | HIC | Health economics analysis | Urban | Midwife-led continuity | Antenatal, intrapartum, postpartum | Midwives |  | Cost analysis | Hospital based | Peer reviewed paper |
| yes | Kildea 2016^90^ | Australia | HIC | Quantitative | Remote | Midwifery group practice | Antenatal, intrapartum, postpartum | Midwives, Aboriginal Health Worker | Indigenous women | Women's outcomes | Hospital based | Peer reviewed paper |
| yes | Kildea 2018^91^ | Australia | HIC | Survey study | Urban | Midwifery group practice | Antenatal, intrapartum, postpartum | Midwives |  | Women's experiences | Hospital based | Peer reviewed paper |
| yes | Kildea 2019^92^ | Australia | HIC | Quantitative | Urban | Midwifery group practice | Antenatal, intrapartum, postpartum | Midwives, Aboriginal Health Worker | Indigenous women | Women's outcomes | Hospital based | Peer reviewed paper |
| yes | Lack 2016^93^ | Australia | HIC | Quantitative | Remote | Midwifery group practice | Antenatal, intrapartum, postpartum | Midwives |  | Women's outcomes | Community | Peer reviewed paper |
| yes | Lang 2019^94^ | United Kingdom | HIC | Quantitative | Urban | Midwife-led continuity | Antenatal, intrapartum, postpartum | Midwives |  | Women's outcomes | Hospital based | Peer reviewed paper |
| yes | Larsson 2020^95^ | Sweden | HIC | Qualitative study | Rural | Caseload midwifery | Antenatal, intrapartum, postpartum | Midwives |  | Women's experiences | Hospital based | Peer reviewed paper |
| yes | Larsson 2021^96^ | Sweden | HIC | Qualitative study | Rural | Caseload midwifery | Antenatal, intrapartum, postpartum | Midwives |  | Midwives experiences | Hospital based | Peer reviewed paper |
| yes | Larsson 2021^97^ | Sweden | HIC | Qualitative study | Rural | Caseload midwifery | Antenatal, intrapartum, postpartum | Midwives |  | Partners experiences | Hospital based | Peer reviewed paper |
| yes | Lewis 2020^98^ | United Kingdom | HIC | Qualitative study | Urban | Caseload midwifery | Antenatal, intrapartum, postpartum | Midwives |  | Midwives experiences | Hospital based | Peer reviewed paper |
| yes | Liambila 2013^99^ | Kenya | LMIC | Qualitative study | Rural | Midwife-led continuity | Antenatal, intrapartum | Midwives |  | Model of care implementation | Community | Peer reviewed paper |
| yes | Liu 2021^100^ | China | LMIC | Survey study | Urban | Midwife-led continuity | Antenatal, intrapartum, postpartum | Midwives |  | Women's experiences | Hospital based | Peer reviewed paper |
| yes | Macfarlane 2014^101^ | United Kingdom | HIC | Survey study | Urban | Midwife-led continuity | Antenatal, intrapartum, postpartum | Midwives |  | Women's experiences | Birth centre | Peer reviewed paper |
| yes | Macmillan 2019^102^ | Australia | HIC | Practice story | Remote | Midwifery group practice | Antenatal, intrapartum, postpartum | Midwives |  | Midwives experiences | Hospital based | Conference Abstract |
| yes | Maillefer 2015^103^ | Switzerland | HIC | Qualitative study | Urban | Midwife-led continuity | Antenatal, intrapartum, postpartum | Midwives |  | Women's experiences | Hospital based | Peer reviewed paper |
| yes | Maimburg 2018^104^ | Denmark | HIC | Quantitative | Urban | Caseload midwifery | Antenatal, intrapartum, postpartum | Midwives |  | Women's outcomes | Hospital based | Peer reviewed paper |
| yes | Markesjö, 2019^105^ | Sweden | HIC | Qualitative study | Urban | Midwifery group practice | Antenatal, intrapartum | Midwives |  | Women's experiences | Hospital based | Published report |
| yes | McCalman, 2020^106^ | Australia | HIC | Qualitative study | Unknown | Caseload midwifery | Antenatal, intrapartum, postpartum | Midwives | Indigenous women | Women's experiences | Hospital based | Conference Abstract |
| yes | McInnes 2020^107^ | United Kingdom | HIC | Qualitative study | Urban | Midwife-led continuity | Antenatal, intrapartum, postpartum | Midwives |  | Model of care implementation | Hospital based | Peer reviewed paper |
| yes | McKellar 2013^108^ | Australia | HIC | Practice story | Both | Caseload midwifery | Antenatal, intrapartum, postpartum | Midwifery students |  | Midwifery students | N/A | Conference Abstract |
| yes | McLachlan 2012^109^ | Australia | HIC | Trial | Urban | Caseload midwifery | Antenatal, intrapartum, postpartum | Midwives |  | Women's outcomes | Hospital based | Peer reviewed paper |
| no | McLachlan 2016^110^  Trial of McLachlan 2012 | Australia | HIC | Trial | Urban | Caseload midwifery | Antenatal, intrapartum, postpartum | Midwives |  | Women's outcomes | Hospital based | Peer reviewed paper |
| no | Forster2016^111^  Trial of McLachlan 2012 | Australia | HIC | Trial | Urban | Caseload midwifery | Antenatal, intrapartum, postpartum | Midwives |  | Women's experiences | Hospital based | Peer reviewed paper |
| no | Davey 2012^112^  Trial of McLachlan 2012 | Australia | HIC | Trial | Urban | Caseload midwifery | Antenatal, intrapartum, postpartum | Midwives |  | Women's outcomes | Hospital based | Peer reviewed paper |
| no | Davey 2015^113^  Trial of McLachlan 2012 | Australia | HIC | Trial | Urban | Caseload midwifery | Antenatal, intrapartum, postpartum | Midwives |  | Women's outcomes | Hospital based | Conference Abstract |
| no | Davey 2013^114^  Trial of McLachlan 2012 | Australia | HIC | Trial | Urban | Caseload midwifery | Antenatal, intrapartum, postpartum | Midwives |  | Women's outcomes | Hospital based | Conference Abstract |
| yes | McRae 2019^115^ | Canada | HIC | Quantitative | Urban | Midwife-led continuity | Antenatal, intrapartum, postpartum | Midwives |  | Women's outcomes | Community | Peer reviewed paper |
| yes | Menke 2014^116^ | Australia | HIC | Qualitative study | Urban | Midwifery group practice | Antenatal, intrapartum, postpartum | Midwives | Socially disadvantaged or vulnerable women | Midwives experiences | Hospital based | Peer reviewed paper |
| yes | Miller, 2020^117^ | New Zealand | HIC | Qualitative study | Unknown | Caseload midwifery | Antenatal, intrapartum, postpartum | Midwives |  | Model of care implementation | Hospital based | Conference Abstract |
| yes | Monk 2014^118^ | Australia | HIC | Quantitative | Urban | Midwifery group practice | Antenatal, intrapartum, postpartum | Midwives |  | Women's outcomes | Hospital based | Peer reviewed paper |
| yes | Mortensen 2018^119^ | Palestine | LMIC | Quantitative | Rural | Midwife-led continuity | Antenatal, intrapartum, postpartum | Midwives |  | Women's outcomes | Hospital based | Peer reviewed paper |
| yes | Mortensen 2019^120^ | Palestine | LMIC | Quantitative | Rural | Midwife-led continuity | Antenatal, intrapartum, postpartum | Midwives |  | Women's outcomes | Hospital based | Peer reviewed paper |
| yes | Mortensen 2019^121^ | Palestine | LMIC | Quantitative | Rural | Midwife-led continuity | Antenatal, intrapartum, postpartum | Midwives |  | Midwives experiences | Hospital based | Peer reviewed paper |
| yes | Newton 2016^122^ | Australia | HIC | Qualitative study | Urban | Caseload midwifery | Antenatal, intrapartum, postpartum | Midwives |  | Midwives experiences | Hospital based | Peer reviewed paper |
| yes | Newton 2021^123^ | Australia | HIC | Survey study | Both | Caseload midwifery | Antenatal, intrapartum, postpartum | Midwifery students |  | Midwifery students | Hospital based | Peer reviewed paper |
| yes | Newton 2021^124^ | Australia | HIC | Survey study | Both | Caseload midwifery | Antenatal, intrapartum, postpartum | Midwives |  | Midwives experiences | Hospital based | Peer reviewed paper |
| yes | Offerhaus 2020^125^ | Netherlands | HIC | Quantitative | Urban | Caseload midwifery | Antenatal, intrapartum, postpartum | Midwives |  | Women's outcomes | Community | Peer reviewed paper |
| yes | Perdok 2018^126^ | Netherlands | HIC | Survey study | Urban | Caseload midwifery | Antenatal, intrapartum, postpartum | Midwives |  | Women's experiences | Community | Peer reviewed paper |
| yes | Pullon 2014^127^ | New Zealand | HIC | Qualitative study | Urban | Caseload midwifery | Antenatal, intrapartum, postpartum | Midwives | Socially disadvantaged or vulnerable women | Midwives experiences | Community | Peer reviewed paper |
| yes | Rahman, 2021^128^ | Bangladesh | LMIC | Practice story | Rural | Midwife-led continuity | Antenatal, intrapartum, postpartum | Midwives |  | Model of care implementation | Birth centre | Published report |
| yes | Rayment-Jones 2015^129^ | United Kingdom | HIC | Quantitative | Urban | Caseload midwifery | Antenatal, intrapartum, postpartum | Midwives | Socially disadvantaged or vulnerable women | Women's outcomes | Hospital based | Peer reviewed paper |
| yes | Rayment-Jones 2020^130^ | United Kingdom | HIC | Qualitative study | Urban | Caseload midwifery | Antenatal, intrapartum, postpartum | Midwives | Socially disadvantaged or vulnerable women | Midwives experiences | Hospital based | Peer reviewed paper |
| yes | Rayment-Jones 2021^131^ | United Kingdom | HIC | Quantitative | Urban | Caseload midwifery | Antenatal, intrapartum, postpartum | Midwives | Socially disadvantaged or vulnerable women | Women's outcomes | Hospital based | Peer reviewed paper |
| yes | Reszel 2021^132^ | Canada | HIC | Survey study | Urban | Midwife-led continuity | Antenatal, intrapartum, postpartum | Midwives |  | Women's experiences | Birth centre | Peer reviewed paper |
| yes | Rocca-Ihenacho 2021^133^ | United Kingdom | HIC | Qualitative study | Urban | Midwife-led continuity | Antenatal, intrapartum, postpartum | Midwives |  | Model of care implementation | Hospital based | Peer reviewed paper |
| yes | Rosyidah, 2018^134^ | Indonesia | LMIC | Survey study | Urban | Midwife-led continuity | Antenatal, intrapartum, postpartum | Midwifery students |  | Women's experiences | Hospital based | Peer reviewed paper |
| yes | Saleem 2015^135^ | Pakistan | LMIC | Qualitative study | Urban | Midwife-led continuity | Antenatal, intrapartum, postpartum | Midwives |  | Midwives experiences | Hospital based | Peer reviewed paper |
| yes | Scholz, 2019^136^ | Germany | HIC | Survey study | Urban | Midwife-led continuity | Antenatal, intrapartum, postpartum | Midwives |  | Midwives experiences | Hospital based | Unpublished report |
| yes | Shahinfar 2021^137^ | Iran | LMIC | Qualitative study | Urban | Team midwifery | Antenatal, intrapartum, postpartum | Midwives |  | Women's experiences | Hospital based | Peer reviewed paper |
| yes | Sidebotham 2019^138^ | Australia | HIC | Qualitative study | Urban | Caseload midwifery | Antenatal, intrapartum, postpartum | Midwifery students |  | Midwifery students | Hospital based | Peer reviewed paper |
| yes | Simcock 2018^139^ | Australia | HIC | Quantitative | Urban | Midwifery group practice | Antenatal, intrapartum, postpartum | Midwives |  | Women's experiences | Hospital based | Peer reviewed paper |
| yes | Sioti, 2020^140^ | European Union | HIC | Qualitative study | Urban | Midwife-led continuity | Antenatal, intrapartum, postpartum | Midwives |  | Model of care implementation | Community | Conference Abstract |
| yes | Smits, 2020^141^ | Australia | HIC | Practice story | Rural | Midwifery group practice | Antenatal, intrapartum, postpartum | Midwives |  | Model of care implementation | Hospital based | Conference Abstract |
| yes | Stanton 2019^142^ | Australia | HIC | Practice story | Urban | Midwifery group practice | Antenatal, intrapartum, postpartum | Midwives |  | Model of care implementation | Hospital based | Conference Abstract |
| yes | Styles 2020^143^ | Australia | HIC | Qualitative study | Urban | Caseload midwifery | Antenatal, intrapartum, postpartum | Midwives |  | Midwives experiences | Hospital based | Peer reviewed paper |
| yes | Symon 2020^144^ | United Kingdom | HIC | Quantitative | Urban | Midwife-led continuity | Antenatal, intrapartum, postpartum | Midwives |  | Women's outcomes | Community | Peer reviewed paper |
| yes | Symon 2020^145^ | United Kingdom | HIC | Qualitative study | Urban | Midwife-led continuity | Antenatal, intrapartum, postpartum | Midwives |  | Midwives experiences | Community | Peer reviewed paper |
| yes | Synergy Health and Business Collaborative, 2021^146^ | Australia | HIC | Practice story | Urban | Midwifery group practice | Antenatal, postpartum | Midwives |  | Model of care implementation | Community | Published report |
| yes | Taylor 2019^147^ | United Kingdom | HIC | Survey study | Urban | Caseload midwifery | Antenatal, intrapartum, postpartum | Midwives |  | Midwives experiences | Hospital based | Peer reviewed paper |
| yes | Thommesen 2020^148^ | Afghanistan | LMIC | Qualitative study | Rural | Midwife-led clinic | Antenatal, intrapartum, postpartum | Midwives |  | Women's experiences | Community | Peer reviewed paper |
| yes | Tickle 2021^149^ | Australia | HIC | Quantitative | Urban | Caseload midwifery | Antenatal, intrapartum, postpartum | Midwifery students |  | Midwifery students | Hospital based | Peer reviewed paper |
| yes | Tickle 2021^150^ | Australia | HIC | Survey study | Urban | Caseload midwifery | Antenatal, intrapartum, postpartum | Midwifery students |  | Midwifery students | Hospital based | Peer reviewed paper |
| yes | Tietjen 2021^151^ | Germany | HIC | Quantitative | Urban | Midwife-led continuity | Antenatal, intrapartum, postpartum | Midwives |  | Women's experiences | Birth centre | Peer reviewed paper |
| yes | Toohill 2012^152^ | Australia | HIC | Health economics analysis | Urban | Midwifery group practice | Antenatal, intrapartum, postpartum | Midwives |  | Cost analysis | Hospital based | Peer reviewed paper |
| yes | Tracy 2013^153^ | Australia | HIC | Trial | Urban | Midwifery group practice | Antenatal, intrapartum, postpartum | Midwives |  | Women's outcomes | Hospital based | Peer reviewed paper |
| no | Allen 2019^154^  Trial of Tracy 2013 | Australia | HIC | Trial | Urban | Midwifery group practice | Antenatal, intrapartum, postpartum | Midwives |  | Women's experiences | Hospital based | Peer reviewed paper |
| no | Allen 2017^155^  Trial of Tracy 2013 | Australia | HIC | Trial | Urban | Midwifery group practice | Antenatal, intrapartum, postpartum | Midwives |  | Women's experiences | Hospital based | Peer reviewed paper |
| no | Allen 2020^156^  Trial of Tracy 2013 | Australia | HIC | Trial | Urban | Midwifery group practice | Antenatal, intrapartum, postpartum | Midwives |  | Women's experiences | Hospital based | Peer reviewed paper |
| yes | Tracy 2014^157^ | Australia | HIC | Quantitative | Urban | Midwifery group practice | Antenatal, intrapartum, postpartum | Midwives |  | Women's outcomes | Hospital based | Peer reviewed paper |
| yes | Tran 2017^158^ | Australia | HIC | Quantitative | Rural | Collaborative team | Antenatal, intrapartum, postpartum | Midwives, GPs |  | Model of care implementation | Hospital based | Peer reviewed paper |
| yes | Tuominen 2012^159^ | Finland | HIC | Survey study | Urban | Primary maternity care | Antenatal, postpartum | Public health nurse |  | Women's experiences | Community | Peer reviewed paper |
| yes | Turner 2020^160^ | Ireland | HIC | Quantitative | Urban | Private obstetric care | Antenatal, intrapartum, postpartum | Obstetricians |  | Women's outcomes | Hospital based | Peer reviewed paper |
| yes | Turner 2021^161^ | United Kingdom | HIC | Qualitative study | Urban | Midwife-led continuity | Antenatal, intrapartum, postpartum | Midwives |  | Managers | Hospital based | Peer reviewed paper |
| yes | Van Wagner 2012^162^ | Canada | HIC | Quantitative | Remote | Midwife-led continuity | Antenatal, intrapartum, postpartum | Midwives | Indigenous women | Women's outcomes | Birth centre | Peer reviewed paper |
| yes | Van Wagner, 2021^163^ | Canada | HIC | Practice story | Urban | Midwife-led continuity | Antenatal, intrapartum, postpartum | Midwives |  | Model of care implementation | Community | Conference Abstract |
| yes | Vasilevski 2021^164^ | Australia | HIC | Survey study | Urban | Midwifery group practice | Antenatal, intrapartum, postpartum | Midwives |  | Women's experiences | Hospital based | Peer reviewed paper |
| yes | Viveiros 2018^165^ | Canada | HIC | Qualitative study | Urban | Midwife-led continuity | Antenatal, intrapartum, postpartum | Midwives |  | Women's experiences | Community | Peer reviewed paper |
| yes | Voon 2017^166^ | Singapore | HIC | Quantitative | Urban | Caseload midwifery | Antenatal, intrapartum, postpartum | Midwives |  | Women's outcomes | Hospital based | Peer reviewed paper |
| yes | Weisband 2018^167^ | USA | HIC | Quantitative | Urban | Midwife-led continuity | Antenatal, intrapartum, postpartum | Midwives |  | Women's outcomes | Hospital based | Peer reviewed paper |
| yes | Welffens 2020^168^ | Belgium | HIC | Quantitative | Urban | Midwife-led continuity | Antenatal, intrapartum, postpartum | Midwives, obstetricians |  | Women's outcomes | Birth centre | Peer reviewed paper |
| yes | Wernham, 2016^169^ | New Zealand | HIC | Quantitative | Both | Caseload midwifery | Antenatal, intrapartum, postpartum | Midwives |  | Women's outcomes | Hospital based | Peer reviewed paper |
| yes | West 2016^170^ | Australia | HIC | Qualitative study | Both | Caseload midwifery | Antenatal, intrapartum, postpartum | Midwifery students | Indigenous women | Midwifery students | Hospital based | Peer reviewed paper |
| yes | Westbury, 2020^171^ | United Kingdom | HIC | Practice story | Urban | Midwife-led continuity | Antenatal, intrapartum, postpartum | Midwives |  | Model of care implementation | Community | Conference Abstract |
| yes | Wilkes 2015^172^ | Australia | HIC | Quantitative | Both | Caseload midwifery - private | Antenatal, intrapartum, postpartum | Midwives |  | Women's outcomes | Community | Peer reviewed paper |
| yes | Wong 2015^173^ | Australia | HIC | Quantitative | Urban | Midwifery group practice | Antenatal, intrapartum, postpartum | Midwives |  | Women's outcomes | Hospital based | Peer reviewed paper |
| yes | Yanti 2015^174^ | Indonesia | LMIC | Survey study | Urban | Midwife-led continuity | Antenatal, intrapartum, postpartum | Midwifery students |  | Midwifery students | Hospital based | Peer reviewed paper |

# Quantitative includes cohort, cross sectional, descriptive, observational studies

**References**

1. Allen J, Gibbons K, Beckmann M, et al. Does model of maternity care make a difference to birth outcomes for young women? A retrospective cohort study. *International Journal of Nursing Studies* 2015;52(8):1332-42. doi: 10.1016/j.ijnurstu.2015.04.011

2. Allen J, Kildea S, Stapleton H. How does group antenatal care function within a caseload midwifery model? A critical ethnographic analysis. *Midwifery* 2015;31(5):489-97. doi: 10.1016/j.midw.2015.01.009

3. Allen J, Kildea S, Stapleton H. How optimal caseload midwifery can modify predictors for preterm birth in young women: Integrated findings from a mixed methods study. *Midwifery* 2016;41:30-38. doi: 10.1016/j.midw.2016.07.012

4. Anwar S, Jan R, Qureshi RN, et al. Perinatal women's perceptions about midwifery led model of care in secondary care hospitals in Karachi, Pakistan. *Midwifery* 2014;30(3):e79-90. doi: 10.1016/j.midw.2013.10.020 [published Online First: 2013/12/03]

5. Aune I, Dahlberg Msc U, Ingebrigtsen O. Parents' experiences of midwifery students providing continuity of care. *Midwifery* 2012;28(4):372-8. doi: https://dx.doi.org/10.1016/j.midw.2011.06.006

6. Aune I, Dahlberg U, Backe B, et al. Comparing standard maternity care with team midwifery care provided by student midwives -- a pilot study. *Nordic Journal of Nursing Research & Clinical Studies / Vård i Norden* 2013;33(3):14-19. doi: 10.1177/010740831303300304

7. Aune I, Tysland T, Amalie Vollheim S. Norwegian midwives' experiences of relational continuity of midwifery care in the primary healthcare service: A qualitative descriptive study. *Nordic Journal of Nursing Research* 2021;41(1):5-13. doi: 10.1177/2057158520973202

8. Baird K, Hastie C, Stanton P, et al. Learning to be a midwife: Midwifery students' experiences of an extended placement within a midwifery group practice. *Women and Birth* 2021 doi: 10.1016/j.wombi.2021.01.002

9. Barimani M, Hylander I. Joint action between child health care nurses and midwives leads to continuity of care for expectant and new mothers. *International Journal of Qualitative Studies on Health and Well being* 2012;7 doi: https://dx.doi.org/10.3402/qhw.v7i0.18183

10. Barker I, Steventon A, Deeny S. Association between continuity of care in general practice and hospital admissions for ambulatory care sensitive conditions: cross sectional study of routinely collected, person level data. *BMJ* 2017;356:j84. doi: 10.1136/bmj.j84

11. Beake S, Acosta L, Cooke P, et al. Caseload midwifery in a multi-ethnic community: the women's experiences. *Midwifery* 2013;29(8):996-1002. doi: https://dx.doi.org/10.1016/j.midw.2013.01.003

12. Beckmann M, Kildea S, Gibbons K. Midwifery group practice and mode of birth. *Women and Birth* 2012;25(4):187-93. doi: https://pubmed.ncbi.nlm.nih.gov/22169396/

13. Biswas A, Anderson R, Doraiswamy S, et al. Timely referral saves the lives of mothers and newborns: midwifery led continuum of care in marginalized teagarden communities - a qualitative case study in Bangladesh. *F1000Research* 2018;7:365. doi: 10.12688/f1000research.13605.1

14. Brintworth K, Sandall J. What makes a successful home birth service: an examination of the influential elements by review of one service. *Midwifery* 2013;29(6):713-21. doi: https://dx.doi.org/10.1016/j.midw.2012.06.016

15. Browne J, Taylor J. 'It's a good thing...': Women's views on their continuity experiences with midwifery students from one Australian region. *Midwifery* 2014;30(3):E108-E14. doi: 10.1016/j.midw.2013.11.006

16. Burau V, Overgaard C. Caseload midwifery as organisational change: the interplay between professional and organisational projects in Denmark. *BMC Pregnancy and Childbirth* 2015;15 doi: 10.1186/s12884-015-0546-8

17. Butcher A, Williams P, Jones F. Is the introduction of a named midwife for teenagers associated with improved outcomes? A service development project. *British Journal of Midwifery* 2016;24(5):331-38. doi: 10.12968/bjom.2016.24.5.331

18. Butska L, Stoll K. Continuity of care, financial stress, and midwifery wellbeing: a comparison of survey results from two Canadian provinces - ICM Congress 2021. The Hague: International Confederation of Midwives 2021.

19. Callander EJ, Slavin V, Gamble J, et al. Cost-effectiveness of public caseload midwifery compared to standard care in an Australian setting: A pragmatic analysis to inform service delivery. *International Journal for Quality in Health Care* 2021;33(2) (no pagination)

20. Carter AG, Wilkes E, Gamble J, et al. Midwifery students' experiences of an innovative clinical placement model embedded within midwifery continuity of care in Australia. *Midwifery* 2015;31(8):765-71. doi: https://dx.doi.org/10.1016/j.midw.2015.04.006

21. Churchill ME, Smylie JK, Wolfe SH, et al. Conceptualising cultural safety at an Indigenous-focused midwifery practice in Toronto, Canada: qualitative interviews with Indigenous and non-Indigenous clients. *BMJ Open* 2020;10(9):e038168. doi: https://dx.doi.org/10.1136/bmjopen-2020-038168

22. Collins M, Kingdon C. One to One midwives: First-year outcomes of a midwifery-led model. *British Journal of Midwifery* 2014;22(1):15-21.

23. Cummins AM, Denney-Wilson E, Homer CS. The experiences of new graduate midwives working in midwifery continuity of care models in Australia. *Midwifery* 2015;31(4):438-44. doi: https://dx.doi.org/10.1016/j.midw.2014.12.013

24. Cummins A, Griew K, Devonport C, et al. Exploring the value and acceptability of an antenatal and postnatal midwifery continuity of care model to women and midwives, using the Quality Maternal Newborn Care Framework. *Women and Birth* 2021 doi: 10.1016/j.wombi.2021.03.006

25. Dahlberg U, Haugan G, Aune I. Women's experiences of home visits by midwives in the early postnatal period. *Midwifery* 2016;39:57-62. doi: https://dx.doi.org/10.1016/j.midw.2016.05.003

26. Daigle K, Hatem M. Co-construct a model of relational continuity in perinatality with women (ICM Abstract 1829). The Hague: International Confederation of Midwives 2021.

27. Davison C, Hauck YL, Bayes SJ, et al. The relationship is everything: Women's reasons for choosing a privately practising midwife in Western Australia. *Midwifery* 2015;31(8):772-8. doi: https://dx.doi.org/10.1016/j.midw.2015.04.012

28. Dawson K, McLachlan H, Newton M, et al. Implementing caseload midwifery: Exploring the views of maternity managers in Australia - A national cross-sectional survey. *Women and Birth* 2016;29(3):214-22. doi: https://dx.doi.org/10.1016/j.wombi.2015.10.010

29. Dawson K, Forster DA, McLachlan HL, et al. Operationalising caseload midwifery in the Australian public maternity system: Findings from a national cross-sectional survey of maternity managers. *Women and Birth* 2018;31(3):194-201. doi: https://dx.doi.org/10.1016/j.wombi.2017.08.132

30. de Jonge A, Stuijt R, Eijke I, et al. Continuity of care: what matters to women when they are referred from primary to secondary care during labour? a qualitative interview study in the Netherlands. *BMC Pregnancy and Childbirth* 2014;14(1):103. doi: 10.1186/1471-2393-14-103

31. Denham S, Humphrey T, Taylor R. Quality of care provided in two Scottish rural community maternity units: a retrospective case review. *BMC Pregnancy & Childbirth* 2017;17(1):198. doi: https://dx.doi.org/10.1186/s12884-017-1374-9

32. Dery S, Aikins M, Maya E. Longitudinal continuity of care during antenatal and delivery in the Volta Region of Ghana. *International Journal of Gynecology & Obstetrics* 2020;151(2):219-24. doi: 10.1002/ijgo.13301

33. de Wolff MG, Midtgaard J, Johansen M, et al. Effects of a Midwife-Coordinated Maternity Care Intervention (ChroPreg) vs. Standard Care in Pregnant Women with Chronic Medical Conditions: Results from a Randomized Controlled Trial. *International Journal of Environmental Research & Public Health [Electronic Resource]* 2021;18(15):25. doi: https://dx.doi.org/10.3390/ijerph18157875

34. Dharni N, Essex H, Bryant MJ, et al. The key components of a successful model of midwifery-led continuity of carer, without continuity at birth: findings from a qualitative implementation evaluation. *BMC Pregnancy & Childbirth* 2021;21(1):205. doi: https://dx.doi.org/10.1186/s12884-021-03671-2

35. Dixon L, Guilliland K, Pallant J, et al. The emotional wellbeing of New Zealand midwives: Comparing responses for midwives in caseloading and shift work settings. *New Zealand College of Midwives Journal* 2017(53):5-14. doi: 10.12784/nzcomjnl53.2017.1.5-14

36. Dobbs K, Turner S, Hill M. Meeting the needs of rural women: an innovative midwifery model of care (ICM Abstract 0648): International Confederation of Midwives 2021.

37. Doering K, Gilkison A, McAra-Couper J. Exploring woman–midwife relationships to enhance women’s positive birth experience in Japan. The Hague: International Confederation of Midwives 2021.

38. Durst M, Rolfe M, Longman J, et al. Local birthing services for rural women: Adaptation of a rural New South Wales maternity service. *Australian Journal of Rural Health* 2016;24(6):385-91. doi: https://dx.doi.org/10.1111/ajr.12310

39. Edmondson MC, Walker SB. Working in caseload midwifery care: the experience of midwives working in a birth centre in North Queensland. *Women and Birth* 2014;27(1):31-6. doi: https://dx.doi.org/10.1016/j.wombi.2013.09.003

40. Fenwick J, Brittain H, Gamble J. Australian private midwives with hospital visiting rights in Queensland: Structures and processes impacting clinical outcomes. *Women and Birth* 2017;30(6):497-505. doi: https://dx.doi.org/10.1016/j.wombi.2017.05.001

41. Fenwick J, Sidebotham M, Gamble J, et al. The emotional and professional wellbeing of Australian midwives: A comparison between those providing continuity of midwifery care and those not providing continuity. *Women and Birth* 2018;31(1):38-43. doi: https://dx.doi.org/10.1016/j.wombi.2017.06.013

42. Fernandez Turienzo C, Silverio SA, Coxon K, et al. Experiences of maternity care among women at increased risk of preterm birth receiving midwifery continuity of care compared to women receiving standard care: Results from the POPPIE pilot trial. *PLoS ONE [Electronic Resource]* 2021;16(4):e0248588. doi: https://dx.doi.org/10.1371/journal.pone.0248588

43. Turienzo CF, Bick D, Briley AL, et al. Midwifery continuity of care versus standard maternity care for women at increased risk of preterm birth: A hybrid implementation-effectiveness, randomised controlled pilot trial in the UK. *PLOS Medicine* 2020;17(10) (no pagination)

44. Flora K, Blandthorn J. A midwifery case management model for women with complex substance use in pregnancy. *Women and Birth* 2018;31:S8-S9. doi: 10.1016/j.wombi.2018.08.034

45. Floris L, Irion O, Bonnet J, et al. Comprehensive maternity support and shared care in Switzerland: Comparison of levels of satisfaction. *Women and Birth* 2018;31(2):124-33. doi: 10.1016/j.wombi.2017.06.021

46. Foster W, Sweet L, Graham K. Midwifery students experience of continuity of care: A mixed methods study. *Midwifery* 2021;98:102966. doi: https://dx.doi.org/10.1016/j.midw.2021.102966

47. Fox D, Chu L, Kelly E, et al. One-to-one midwifery care in Singapore -- the first 100 births. *British Journal of Midwifery* 2013;21(10):701-07.

48. Fujita K, et al. Policies and management of midwife-led unit: Questionnaire-based interview survey. *Journal of Japan Academy of Midwifery* 2018;32:147-58.

49. Gao Y, Gold L, Josif C, et al. A cost-consequences analysis of a midwifery group practice for Aboriginal mothers and infants in the top end of the Northern Territory, Australia. *Midwifery* 2014;30(4):447-55. doi: https://dx.doi.org/10.1016/j.midw.2013.04.004

50. Gidaszewski B, Khajehei M, Gibbs E, et al. Comparison of the effect of caseload midwifery program and standard midwifery-led care on primiparous birth outcomes: A retrospective cohort matching study. *Midwifery* 2019;69:10-16. doi: 10.1016/j.midw.2018.10.010

51. Gilkison A, McAra-Couper J, Gunn J, et al. Midwifery practice arrangements which sustain caseloading Lead Maternity Carer midwives in New Zealand. *New Zealand College of Midwives Journal* 2015(51):11-16. doi: 10.12784/nzcomjnl51.2015.2.11-16

52. Goodman D. Improving Access to Maternity Care for Women with Opioid Use Disorders: Colocation of Midwifery Services at an Addiction Treatment Program. *Journal of Midwifery & Womens Health* 2015;60(6):706-12. doi: 10.1111/jmwh.12340

53. Gray JE, Leap N, Sheehy A, et al. The 'follow-through' experience in three-year Bachelor of Midwifery programs in Australia: a survey of students. *Nurse Education in Practice* 2012;12(5):258-63. doi: https://dx.doi.org/10.1016/j.nepr.2012.04.013

54. Gray J, Leap N, Sheehy A, et al. Students' perceptions of the follow-through experience in 3 year bachelor of midwifery programmes in Australia. *Midwifery* 2013;29(4):400-6. doi: https://dx.doi.org/10.1016/j.midw.2012.07.015

55. Griffiths C, McAra-Couper J, Nayar S. Staying Involved "Because the Need Seems So Huge": Midwives Working With Women Living in Areas of High Deprivation. *International Journal of Childbirth* 2013;3(4):218-31. doi: 10.1891/2156-5287.3.4.218

56. Gross M, Michelsen C, Vaske B, et al. Intrapartum Care Working Patterns of Midwives: The Long Road to Models of Care in Germany. *Z Geburtsh Neonatol* 2017;221:1–10.

57. Grylka-Baeschlin S, Borner B, Pehlke-Milde J. Occupational Situation of Midwives in Institutions with and without Midwife-led Care in in a Swiss Canton. *Zeitschrift fur Geburtshilfe und Neonatologie* 2020;224(2):93-102. doi: 10.1055/a-1083-7028

58. Gu CWXDYZXZZ. The effectiveness of a Chinese midwives' antenatal clinic service on childbirth outcomes for primipare: A randomised controlled trial. *International Journal of Nursing Studies* 2013;50:1689-97. doi: 10.1016/j.ijnurstu.2013.05.001

59. Hadebe R, Seed PT, Essien D, et al. Can birth outcome inequality be reduced using targeted caseload midwifery in a deprived diverse inner city population? A retrospective cohort study, London, UK. *BMJ Open* 2021;11(11):e049991. doi: https://dx.doi.org/10.1136/bmjopen-2021-049991

60. Hailemeskel S, Alemu K, Christensson K, et al. Health care providers' perceptions and experiences related to Midwife-led continuity of care-A qualitative study. *PLoS ONE* 2021;16(10):e0258248. doi: https://dx.doi.org/10.1371/journal.pone.0258248

61. Hailemeskel S, Alemu K, Christensson K, et al. Midwife-led continuity of care improved maternal and neonatal health outcomes in north Shoa zone, Amhara regional state, Ethiopia: A quasi-experimental study. *Women and Birth* 2021 doi: 10.1016/j.wombi.2021.08.008 [published Online First: 2021/09/08]

62. Haines H, Baker J, Marshall D. Continuity of midwifery care for rural women through caseload group practice: Delivering for almost 20 years. *Australian Journal of Rural Health* 2015;23(6):339-45. doi: 10.1111/ajr.12232

63. Hardeman RR, Karbeah J, Almanza J, et al. Roots Community Birth Center: A culturally-centered care model for improving value and equity in childbirth. *Healthcare* 2020;8(1):100367. doi: https://dx.doi.org/10.1016/j.hjdsi.2019.100367

64. Hartz DL, Blain J, Caplice S, et al. Evaluation of an Australian Aboriginal model of maternity care: The Malabar Community Midwifery Link Service. *Women and Birth* 2019;32(5):427-36. doi: https://dx.doi.org/10.1016/j.wombi.2019.07.002

65. Hewitt L, Dadich A, Hartz D, et al. Management and sustainability of midwifery group practice: Thematic and lexical analyses of midwife interviews. *Women and Birth* 2021 doi: 10.1016/j.wombi.2021.05.002

66. Hildingsson I, Rubertsson C, Karlstrom A, et al. Caseload midwifery for women with fear of birth is a feasible option. *Sexual & Reproductive Healthcare* 2018;16:50-55. doi: https://dx.doi.org/10.1016/j.srhc.2018.02.006

67. Hildingsson I, Rubertsson C, Karlström A, et al. A known midwife can make a difference for women with fear of childbirth- birth outcome and women's experiences of intrapartum care. *Sexual & Reproductive HealthCare* 2019;21:33-38. doi: 10.1016/j.srhc.2019.06.004

68. Hildingsson I, Karlstrom A, Rubertsson C, et al. Birth outcome in a caseload study conducted in a rural area of Sweden-a register based study. *Sexual & Reproductive Healthcare* 2020;24:100509. doi: https://dx.doi.org/10.1016/j.srhc.2020.100509

69. Hildingsson I, Karlstrom A, Larsson B. A continuity of care project with two on-call schedules: Findings from a rural area in Sweden. *Sexual & Reproductive Healthcare* 2020;26:100551. doi: https://dx.doi.org/10.1016/j.srhc.2020.100551

70. Hildingsson I. Women's Experiences of Care During Pregnancy in a Continuity of Midwifery Care Project in Rural Sweden. *International Journal of Childbirth* 2021;11(3):131-44. doi: 10.1891/IJCBIRTH-D-20-00048

71. Hildingsson I, Karlstrom A, Larsson B. Childbirth experience in women participating in a continuity of midwifery care project. *Women and Birth* 2021;34(3):e255-e61. doi: https://dx.doi.org/10.1016/j.wombi.2020.04.010

72. Hollins Martin CJ, MacArthur J, Martin CR, et al. Midwives' views of changing to a Continuity of Midwifery Care (CMC) model in Scotland: A baseline survey. *Women and Birth* 2020;33(5):e409-e19. doi: https://dx.doi.org/10.1016/j.wombi.2019.08.005

73. Holroyd M. Midwives experiences of working within the project “Min Barnmorska”. Stockholm: Karolinska Institute 2019.

74. Homer CS, Foureur MJ, Allende T, et al. 'It's more than just having a baby' women's experiences of a maternity service for Australian Aboriginal and Torres Strait Islander families. *Midwifery* 2012;28(4):E449-55. doi: https://dx.doi.org/10.1016/j.midw.2011.06.004

75. Homer C, Leap N, Edwards N, et al. Midwifery continuity of care in an area of high socio-economic disadvantage in London: A retrospective analysis of Albany Midwifery Practice outcomes using routine data (1997-2009). *Midwifery* 2017;48:1-10. doi: 10.1016/j.midw.2017.02.009

76. Homer CSE, Davis DL, Mollart L, et al. Midwifery continuity of care and vaginal birth after caesarean section: A randomised controlled trial. *Women and Birth* 2021

77. Hopkinson D, Kearney L, Gray M, et al. Strength with immersion model (SwIM): new midwives employed within continuity models: evaluation of a Queensland state-wide initiative to strengthen capacity and support organisational change (ICM Congress Abstract). The Hague: International Confederation of Midwives 2021.

78. Hua J, Zhu L, Du L, et al. Effects of midwife-led maternity services on postpartum wellbeing and clinical outcomes in primiparous women under China's one-child policy. *BMC Pregnancy & Childbirth* 2018;18(1):329. doi: https://dx.doi.org/10.1186/s12884-018-1969-9

79. Hunter M, Crowther S, McAra-Couper J, et al. Generosity of spirit sustains caseloading Lead Maternity Carer midwives in New Zealand. *MIDIRS Midwifery Digest* 2017;27(1):32-32.

80. Iida M, Horiuchi S, Porter SE. The relationship between women-centred care and women's birth experiences: a comparison between birth centres, clinics, and hospitals in Japan. *Midwifery* 2012;28(4):398-405. doi: https://dx.doi.org/10.1016/j.midw.2011.07.002

81. Iida M, Horiuchi S, Nagamori K. A comparison of midwife-led care versus obstetrician-led care for low-risk women in Japan. *Women and Birth* 2014;27(3):202-07. doi: 10.1016/j.wombi.2014.05.001

82. Iida M, Horiuchi S, Nagamori K. Women's experience of receiving team-midwifery care in Japan: A qualitative descriptive study. *Women and Birth* 2021;34(5):493-99. doi: 10.1016/j.wombi.2020.09.020

83. Jepsen I, Foureur M, Noehr E, et al. Qualitative research on how midwives experience caseload midwifery. *Women and Birth* 2015;28(1):S48-S48. doi: 10.1016/j.wombi.2015.07.153

84. Jepsen I, Juul S, Foureur M, et al. Is caseload midwifery a healthy work-form? - A survey of burnout among midwives in Denmark. *Sexual & Reproductive Healthcare* 2017;11:102-06. doi: https://dx.doi.org/10.1016/j.srhc.2016.12.001

85. Jepsen I, Juul S, Foureur MJ, et al. Labour outcomes in caseload midwifery and standard care: a register-based cohort study. *BMC Pregnancy & Childbirth* 2018;18(1):481. doi: https://dx.doi.org/10.1186/s12884-018-2090-9

86. Josif CM, Barclay L, Kruske S, et al. 'No more strangers': Investigating the experiences of women, midwives and others during the establishment of a new model of maternity care for remote dwelling Aboriginal women in northern Australia. *Midwifery* 2014;30(3):317-23. doi: https://dx.doi.org/10.1016/j.midw.2013.03.012

87. Kashani A, Ingberg J, Hildingsson I. Caseload midwifery in a rural Australian setting: A qualitative descriptive study. *European Journal of Midwifery* 2021;5:2-2. doi: 10.18332/ejm/131240

88. Kelly J, West R, Gamble J, et al. 'She knows how we feel': Australian Aboriginal and Torres Strait Islander childbearing women's experience of Continuity of Care with an Australian Aboriginal and Torres Strait Islander midwifery student. *Women and Birth* 2014;27(3):157-62. doi: https://pubmed.ncbi.nlm.nih.gov/24997119/

89. Kenny C, Devane D, Normand C, et al. A cost-comparison of midwife-led compared with consultant-led maternity care in Ireland (the MidU study). *Midwifery* 2015;31(11):1032-38. doi: 10.1016/j.midw.2015.06.012

90. Kildea S, Gao Y, Rolfe M, et al. Remote links: Redesigning maternity care for Aboriginal women from remote communities in Northern Australia - A comparative cohort study. *Midwifery* 2016;34:47-57. doi: https://dx.doi.org/10.1016/j.midw.2016.01.009

91. Kildea S, Simcock G, Liu A, et al. Continuity of midwifery carer moderates the effects of prenatal maternal stress on postnatal maternal wellbeing: the Queensland flood study. *Archives of Women's Mental Health* 2018;21(2):203-14. doi: 10.1007/s00737-017-0781-2

92. Kildea S, Gao Y, Hickey S, et al. Reducing preterm birth amongst Aboriginal and Torres Strait Islander babies: A prospective cohort study, Brisbane, Australia. *EClinicalMedicine* 2019;12:43-51. doi: 10.1016/j.eclinm.2019.06.001

93. Lack BM, Smith RM, Arundell MJ, et al. Narrowing the Gap? Describing women's outcomes in Midwifery Group Practice in remote Australia. *Women and Birth* 2016;29(5):465-70. doi: https://dx.doi.org/10.1016/j.wombi.2016.03.003

94. Lang N, Jose S, Rogers A, et al. Better Births: early findings from North-West London. *British Journal of Midwifery* 2019;27(9):555-61. doi: 10.12968/bjom.2019.27.9.555

95. Larsson B, Rubertsson C, Hildingsson I. A modified caseload midwifery model for women with fear of birth, women's and midwives' experiences: A qualitative study. *Sexual & reproductive healthcare : official journal of the Swedish Association of Midwives* 2020;24:100504. doi: https://dx.doi.org/10.1016/j.srhc.2020.100504

96. Larsson B, Thies-Lagergren L, Karlstrom A, et al. Demanding and rewarding: Midwives experiences of starting a continuity of care project in rural Sweden. *European Journal of Midwifery* 2021;5:8-8. doi: 10.18332/ejm/133573

97. Larsson B, Thies-Lagergren L. Partners' expectations and experiences of the project 'Midwife All the Way': A qualitative study. *European Journal of Midwifery* 2021;5:17-17. doi: 10.18332/ejm/136424

98. Lewis M. Midwives' Experience of Providing Continuity of Care in a Pilot Project: Findings of a Prospective Qualitative Research Study. *International Journal of Childbirth* 2020;10(3):151-61. doi: 10.1891/IJCBIRTH-D-20-00009

99. Liambila W, Obare F, Undie C, et al. The community midwifery model in Kenya: Expanding access to comprehensive reproductive health services at the community level. *African Journal of Midwifery and Women's Health* 2013;7(4):171-77. doi: 10.12968/ajmw.2013.7.4.171

100. Liu Y, Li T, Guo N, et al. Women's experience and satisfaction with midwife-led maternity care: a cross-sectional survey in China. *BMC Pregnancy & Childbirth* 2021;21(1):151. doi: https://dx.doi.org/10.1186/s12884-021-03638-3

101. Macfarlane AJ, Rocca-Ihenacho L, Turner LR. Survey of women's experiences of care in a new freestanding midwifery unit in an inner city area of London, England: 2. Specific aspects of care. *Midwifery* 2014;30(9):1009-20. doi: https://dx.doi.org/10.1016/j.midw.2014.05.008

102. Macmillan G, Wood M, Bettison H. Sustainable midwifery continuity on a tropical island where it shouldn't work but it does! *Women and Birth* 2019;32:S25-S25. doi: 10.1016/j.wombi.2019.07.221

103. Maillefer F, de Labrusse C, Cardia-Voneche L, et al. Women and healthcare providers' perceptions of a midwife-led unit in a Swiss university hospital: A qualitative study. *BMC Pregnancy and Childbirth* 2015;15(1) (no pagination) doi: https://pubmed.ncbi.nlm.nih.gov/25886389/

104. Maimburg RD. Homebirth organised in a caseload midwifery model with affiliation to a Danish university hospital - A descriptive study. *Sexual & Reproductive Healthcare* 2018;16:82-85. doi: https://dx.doi.org/10.1016/j.srhc.2018.02.011

105. Markesjö G. Women’s experience of pregnancy and childbirth in a continuity of care model. A qualitative study at Huddinge Hospital. Stockholm: Karolinska Institute 2019.

106. McCalman P, McLachlan H, Forster D, et al. What does our mob think? Exploring the maternity care experiences of Australian Aboriginal and Torres Strait Islander women who were offered continuity of midwife care (ICM Congress Abstract). The Hague: International Confederation of Midwives 2021.

107. McInnes RJ, Aitken-Arbuckle A, Lake S, et al. Implementing continuity of midwife carer - just a friendly face? A realist evaluation. *BMC Health Services Research* 2020;20(1):304. doi: https://dx.doi.org/10.1186/s12913-020-05159-9

108. McKellar L, Licqurish S, Dove S, et al. Call the midwifery student: Facilitating continuity of care experience in education and practice. *Women and Birth* 2013;26(1):S43-S43. doi: 10.1016/j.wombi.2013.08.134

109. McLachlan HL, Forster DA, Davey MA, et al. Effects of continuity of care by a primary midwife (caseload midwifery) on caesarean section rates in women of low obstetric risk: the COSMOS randomised controlled trial. *BJOG* 2012;119(12):1483-92. doi: https://dx.doi.org/10.1111/j.1471-0528.2012.03446.x

110. McLachlan HL, Forster DA, Davey MA, et al. The effect of primary midwife-led care on women's experience of childbirth: results from the COSMOS randomised controlled trial. *BJOG* 2016;123(3):465-74. doi: https://dx.doi.org/10.1111/1471-0528.13713

111. Forster D, McLachlan H, Davey M, et al. Continuity of care by a primary midwife (caseload midwifery) increases women’s satisfaction with antenatal, intrapartum and postpartum care: results from the COSMOS randomised controlled trial. *BMC Pregnancy and Childbirth* 2016;16(1):28. doi: 10.1186/s12884-016-0798-y

112. Davey M-A, McLachlan HL, Forster D, et al. Influence of timing of admission in labour and management of labour on method of birth: Results from a randomised controlled trial of caseload midwifery (COSMOS trial). *Midwifery* 2013;29(12):1297-302. doi: 10.1016/j.midw.2013.05.014

113. Davey M, McLachlan H, Forster D, et al. Risk factors for excessive postpartum blood loss in the COSMOS trial of caseload midwifery. *Women and Birth* 2015;28(1):S46-S46. doi: 10.1016/j.wombi.2015.07.147

114. Davey M, McLachlan H, Forster D. Timing of admission and selected aspects of intrapartum care: Relationship with caesarean section in the COSMOS (Caseload Midwifery) trial. *Women and Birth* 2013;26(1):S3-S3. doi: 10.1016/j.wombi.2013.08.228

115. McRae DN, Muhajarine N, Janssen PA. Improving birth outcomes for women who are substance using or have mental illness: a Canadian cohort study comparing antenatal midwifery and physician models of care for women of low socioeconomic position. *BMC Pregnancy & Childbirth* 2019;19(1):279. doi: https://dx.doi.org/10.1186/s12884-019-2428-y

116. Menke J, Fenwick J, Gamble J, et al. Midwives' perceptions of organisational structures and processes influencing their ability to provide caseload care to socially disadvantaged and vulnerable women. *Midwifery* 2014;30(10):1096-103. doi: https://dx.doi.org/10.1016/j.midw.2013.12.015

117. Miller S, Maude R, Davis D. First birth in Aotearoa New Zealand: does having access to ‘bells and whistles’ influence the likelihood of normal birth? (ICM Congress Abstract) The Hague: International Confederation of Midwives 2021.

118. Monk A, Tracy M, Foureur M, et al. Evaluating Midwifery Units (EMU): a prospective cohort study of freestanding midwifery units in New South Wales, Australia. *BMJ Open* 2014;4(10):e006252. doi: https://dx.doi.org/10.1136/bmjopen-2014-006252

119. Mortensen B, Lukasse M, Diep LM, et al. Can a midwife-led continuity model improve maternal services in a low-resource setting? A non-randomised cluster intervention study in Palestine. *BMJ Open* 2018;8(3):e019568. doi: https://dx.doi.org/10.1136/bmjopen-2017-019568

120. Mortensen B, Lieng M, Diep LM, et al. Improving maternal and neonatal health by a midwife-led continuity model of care - an observational study in one governmental hospital in Palestine. *EClinicalMedicine* 2019;10:84-91. doi: 10.1016/j.eclinm.2019.04.003 [published Online First: 2019/06/14]

121. Mortensen B, Diep LM, Lukasse M, et al. Women's satisfaction with midwife-led continuity of care: an observational study in Palestine. *BMJ Open* 2019;9(11):e030324. doi: https://dx.doi.org/10.1136/bmjopen-2019-030324

122. Newton M, McLachlan H, Forster D, et al. Understanding the 'ork' of caseload midwives: A mixed-methods exploration of two caseload midwifery models in Victoria, Australia. *Women and Birth* 2016;29(3):223-33. doi: 10.1016/j.wombi.2015.10.011

123. Newton M, Faulks F, Bailey C, et al. Continuity of care experiences: A national cross-sectional survey exploring the views and experiences of Australian students and academics. *Women and Birth* 2021 doi: 10.1016/j.wombi.2021.05.009

124. Newton M, Dawson K, Forster D, et al. Midwives' views of caseload midwifery - comparing the caseload and non-caseload midwives' opinions. A cross-sectional survey of Australian midwives. *Women and Birth* 2021;34(1):e47-e56. doi: https://dx.doi.org/10.1016/j.wombi.2020.06.006

125. Offerhaus P, Jans S, Hukkelhoven C, et al. Women's characteristics and care outcomes of caseload midwifery care in the Netherlands: a retrospective cohort study. *BMC Pregnancy & Childbirth* 2020;20(1):517. doi: https://dx.doi.org/10.1186/s12884-020-03204-3

126. Perdok H, Verhoeven C, van Dillen J, et al. Continuity of care is an important and distinct aspect of childbirth experience: findings of a survey evaluating experienced continuity of care, experienced quality of care and women's perception of labor. *BMC Pregnancy and Childbirth* 2018;18 doi: 10.1186/s12884-017-1615-y

127. Pullon S, Gray B, Steinmetz M, et al. Midwifery-led care embedded within primary care: consumer satisfaction with a model in New Zealand. *Journal of Primary Health Care* 2014;6(4):319-23.

128. Rahman S. Midwife led Care Centre in a Government Facility: Charikata Union Health and Family Welfare Centre (UH&FWC), Jaintiapur, Sylhet. 2021 Wednesday, Apr 20, 2022.

129. Rayment-Jones H, Murrells T, Sandall J. An investigation of the relationship between the caseload model of midwifery for socially disadvantaged women and childbirth outcomes using routine data - A retrospective, observational study. *Midwifery* 2015;31(4):409-17. doi: 10.1016/j.midw.2015.01.003

130. Rayment-Jones H, Silverio SA, Harris J, et al. Project 20: Midwives' insight into continuity of care models for women with social risk factors: what works, for whom, in what circumstances, and how. *Midwifery* 2020;84:102654. doi: https://dx.doi.org/10.1016/j.midw.2020.102654

131. Rayment-Jones H, Dalrymple K, Harris J, et al. Project20: Does continuity of care and community-based antenatal care improve maternal and neonatal birth outcomes for women with social risk factors? A prospective, observational study. *PLoS ONE* 2021;16(5):e0250947. doi: https://dx.doi.org/10.1371/journal.pone.0250947

132. Reszel J, Weiss D, Darling EK, et al. Client Experience with the Ontario Birth Center Demonstration Project. *Journal of Midwifery & Women's Health* 2021;66(2):174-84. doi: https://dx.doi.org/10.1111/jmwh.13164

133. Rocca-Ihenacho L, Yuill C, McCourt C. Relationships and trust: Two key pillars of a well-functioning freestanding midwifery unit. *Birth* 2021;48(1):104-13. doi: https://dx.doi.org/10.1111/birt.12521

134. Rosyidah H, Aisyaroh N. Clients’ satisfaction with continuity of midwifery care. *Jurnal Kesehatan Ibu dan Anak* 2018;12(2):129~35. doi: 10.29238/kia.v12i2.149

135. Saleem Z, Jan R, McInytre H, et al. Midwives' perception about their practice in a midwifery-led care model in Karachi, Pakistan. *British Journal of Midwifery* 2015;23(3):200-07. doi: 10.12968/bjom.2015.23.3.200

136. Scholz G, Dreier M, Gross M. Midwives´ working patterns and scope of midwifery in the Region Hannover: Draft manuscript shared with permission 2021.

137. Shahinfar S, Abedi P, Najafian M, et al. Women's perception of continuity of team midwifery care in Iran: a qualitative content analysis. *BMC Pregnancy Childbirth* 2021;21(1):173. doi: 10.1186/s12884-021-03666-z [published Online First: 2021/03/04]

138. Sidebotham M, Fenwick J. Midwifery students' experiences of working within a midwifery caseload model. *Midwifery* 2019;74:21-28. doi: https://dx.doi.org/10.1016/j.midw.2019.03.008

139. Simcock G, Kildea S, Kruske S, et al. Disaster in pregnancy: midwifery continuity positively impacts infant neurodevelopment, QF2011 study. *BMC Pregnancy & Childbirth* 2018;18(1):309. doi: https://dx.doi.org/10.1186/s12884-018-1944-5

140. Sioti E, Triantafillou E, Soltani H, et al. Process evaluation of the Operational Refugee and Migrant Maternal Approach (ORAMMA), in three different European primary healthcare settings (ICM Congress Abstract) The Hague: International Confederation of Midwives 2021.

141. Smits S. Resistance to change the challenges of starting a midwifery group practice in a small town in outback Queensland - Report on an initiative. First-hand account (ICM Abstract) The Hague: International Confederation of Midwives 2021.

142. Stanton P, Brittain H, Baird K, et al. Expanding a midwifery caseload model within a large tertiary service. *Women and Birth* 2019;32(Supplement 1):S12. doi: https://doi.org/10.1016/j.wombi.2019.07.186

143. Styles C, Kearney L, George K. Implementation and upscaling of midwifery continuity of care: The experience of midwives and obstetricians. *Women and Birth* 2020;33(4):343-51. doi: https://dx.doi.org/10.1016/j.wombi.2019.08.008

144. Symon A, Shinwell S, Craig J. Process lessons from evaluating a combined continuity of carer and home birth scheme. *Birth* 2020;47(4):389-96. doi: https://dx.doi.org/10.1111/birt.12514

145. Symon A, Shinwell S. Qualitative evaluation of an innovative midwifery continuity scheme: Lessons from using a quality care framework. *Birth* 2020;47(4):378-88. doi: 10.1111/birt.12512

146. Synergy Health and Business Collaborative. Logan Community Maternity and Child Health Hubs Cost Analysis. Brisbane: Synergy Health and Business Collaborative 2021.

147. Taylor B, Cross-Sudworth F, Goodwin L, et al. Midwives' perspectives of continuity based working in the UK: A cross-sectional survey. *Midwifery* 2019;75:127-37. doi: https://dx.doi.org/10.1016/j.midw.2019.05.005

148. Thommesen T, Kismul H, Kaplan I, et al. "The midwife helped me ... otherwise I could have died": women's experience of professional midwifery services in rural Afghanistan - a qualitative study in the provinces Kunar and Laghman. *BMC Pregnancy & Childbirth* 2020;20(1):140. doi: https://dx.doi.org/10.1186/s12884-020-2818-1

149. Tickle N, Gamble J, Creedy D. Clinical outcomes for women who had continuity of care experiences with midwifery students. *Women and Birth* 2021 doi: 10.1016/j.wombi.2021.04.004

150. Tickle N, Gamble J, Creedy DK. Women's reports of satisfaction and respect with continuity of care experiences by students: Findings from a routine, online survey. *Women and Birth* 2021;34(6):e592-e98. doi: https://dx.doi.org/10.1016/j.wombi.2020.11.004

151. Tietjen S, Schmitz M, Heep A, et al. Model of care and chance of spontaneous vaginal birth: a prospective, multicenter matched-pair analysis from North Rhine-Westphalia. *BMC Pregnancy and Childbirth* 2021;21(1) doi: 10.1186/s12884-021-04323-1

152. Toohill J, Turkstra E, Gamble J, et al. A non-randomised trial investigating the cost-effectiveness of Midwifery Group Practice compared with standard maternity care arrangements in one Australian hospital. *Midwifery* 2012;28(6):e874-9. doi: https://dx.doi.org/10.1016/j.midw.2011.10.012

153. Tracy S, Hartz D, Tracy M, et al. Caseload midwifery care versus standard maternity care for women of any risk: M@NGO, a randomised controlled trial. *Lancet* 2013;382(9906):1723-32. doi: 10.1016/s0140-6736(13)61406-3

154. Allen J, Kildea S, Tracy M, et al. The impact of caseload midwifery, compared with standard care, on women's perceptions of antenatal care quality: Survey results from the M@NGO randomized controlled trial for women of any risk. *Birth* 2019;46(3):439-49. doi: 10.1111/birt.12436

155. Allen J, Kildea S, Hartz D, et al. The motivation and capacity to go ‘above and beyond’: Qualitative analysis of free-text survey responses in the M@NGO randomised controlled trial of caseload midwifery. *Midwifery* 2017;50:148-56. doi: 10.1016/j.midw.2017.03.012

156. Allen J, Jenkinson B, Tracy S, et al. Women's unmet needs in early labour: Qualitative analysis of free-text survey responses in the M@NGO trial of caseload midwifery. *Midwifery* 2020;88 doi: 10.1016/j.midw.2020.102751

157. Tracy SK, Welsh A, Hall B, et al. Caseload midwifery compared to standard or private obstetric care for first time mothers in a public teaching hospital in Australia: a cross sectional study of cost and birth outcomes. *BMC Pregnancy & Childbirth* 2014;14:46. doi: https://dx.doi.org/10.1186/1471-2393-14-46

158. Tran T, Longman J, Kornelsen J, et al. The development of a caseload midwifery service in rural Australia. *Women and Birth* 2017;30(4):291-97. doi: https://dx.doi.org/10.1016/j.wombi.2016.11.010

159. Tuominen M, Kaljonen A, Ahonen P, et al. Does the organizational model of the maternity health clinic have an influence on women's and their partners' experiences? A service evaluation survey in Southwest Finland. *BMC Pregnancy and Childbirth* 2012;12 doi: 10.1186/1471-2393-12-96

160. Turner M, Reynolds C, McMahon L, et al. Caesarean section rates in women in the Republic of Ireland who chose to attend their obstetrician privately: a retrospective observational study. *BMC Pregnancy and Childbirth* 2020;20(1) doi: 10.1186/s12884-020-03199-x

161. Turner S, Crowther S, Lau A. A grounded theory study on midwifery managers' views and experiences of implementing and sustaining continuity of carer models within the UK maternity system. *Women and Birth* 2021 doi: 10.1016/j.wombi.2021.10.010

162. Van Wagner V, Osepchook C, Harney E, et al. Remote midwifery in Nunavik, Quebec, Canada: outcomes of perinatal care for the Inuulitsivik health centre, 2000-2007. *Birth* 2012;39(3):230-7. doi: https://dx.doi.org/10.1111/j.1523-536X.2012.00552.x

163. Van Wagner V, Brandeis E, Klein L, et al. Supporting midwifery practices to use and share outcomes data (ICM Abstract). The Hague: International Confederation of Midwives 2021.

164. Vasilevski V, Sweet L, Smith L, et al. Part-time positions in Caseload Midwifery Group Practice: Impact on satisfaction and quality of care. *Women and Birth* 2021;34(6):e567-e74. doi: https://dx.doi.org/10.1016/j.wombi.2020.11.001

165. Viveiros C, Darling E. Barriers and facilitators of accessing perinatal mental health services: The perspectives of women receiving continuity of care midwifery. *Midwifery* 2018;65:8-15. doi: 10.1016/j.midw.2018.06.018

166. Voon S, Lay J, San W, et al. Comparison of midwife-led care and obstetrician-led care on maternal and neonatal outcomes in Singapore: A retrospective cohort study. *Midwifery* 2017;53:71-79. doi: 10.1016/j.midw.2017.07.010

167. Weisband YL, Gallo MF, Klebanoff MA, et al. Progression of care among women who use a midwife for prenatal care: Who remains in midwife care? *Birth* 2018;45(1):28-36. doi: https://dx.doi.org/10.1111/birt.12308

168. Welffens K, Derisbourg S, Costa E, et al. The "Cocoon," first alongside midwifery-led unit within a Belgian hospital: Comparison of the maternal and neonatal outcomes with the standard obstetric unit over 2 years. *Birth* 2020;47(1):115-22. doi: https://dx.doi.org/10.1111/birt.12466

169. Wernham E, Gurney J, Stanley J, et al. A Comparison of Midwife-Led and Medical-Led Models of Care and Their Relationship to Adverse Fetal and Neonatal Outcomes: A Retrospective Cohort Study in New Zealand. *PLoS Med* 2016;13(9):e1002134. doi: 10.1371/journal.pmed.1002134 [published Online First: 2016/09/28]

170. West R, Gamble J, Kelly J, et al. Culturally capable and culturally safe: Caseload care for Indigenous women by Indigenous midwifery students. *Women and Birth* 2016;29(6):524-30. doi: https://dx.doi.org/10.1016/j.wombi.2016.05.003

171. Westbury B. Promoting normality and choice; reestablishing a home birth service in North Ceredigion. The Hague: International Confederation of Midwives 2021.

172. Wilkes E, Gamble J, Adam G, et al. Reforming maternity services in Australia: Outcomes of a private practice midwifery service. *Midwifery* 2015;31(10):935-40. doi: https://dx.doi.org/10.1016/j.midw.2015.05.006

173. Wong N, Browne J, Ferguson S, et al. Getting the first birth right: A retrospective study of outcomes for low-risk primiparous women receiving standard care versus midwifery model of care in the same tertiary hospital. *Women and Birth* 2015;28(4):279-84. doi: 10.1016/j.wombi.2015.06.005

174. Yanti Y, Claramita M, Emilia O, et al. Students' understanding of "Women-Centred Care Philosophy" in midwifery care through Continuity of Care (CoC) learning model: a quasi-experimental study. *BMC Nursing* 2015;14:22-22. doi: 10.1186/s12912-015-0072-z
