# Supplementary material for: Midwifery continuity of care: A scoping review of where, how, by whom and for whom?
Source: PLOS Glob Public Health. 2022 Oct 5;2(10):e0000935. doi: 10.1371/journal.pgph.0000935 (PMC10021789; doi:10.1371/journal.pgph.0000935)
Supplement: S2 Table — (DOCX) [file pgph.0000935.s003.docx]

**S2 Table: Additional details from low- and middle-income countries**

**Supplementary file: Additional detail on Midwifery Continuity of Care initiatives in low- and middle-income countries**

*Note: MCoC = Midwifery Continuity of Care*

| **First Author** | **Year published** | **Country** | **Study design** | **Population** | **Setting** | **Model and continuity of care provided** | **Novel initiative or scaled-up** |
| --- | --- | --- | --- | --- | --- | --- | --- |
| Anwar^1^ | 2014 | Pakistan | Qualitative descriptive study (Semi-structured interviews) | 10 women | Urban Karachi.  Two secondary care hospitals sites parallel with conventional medical-led service. | Midwifery led continuity of care service implemented.  Lead midwife providing care across antenatal, intrapartum, postnatal  (although not consistently achieved) | Novel |
| Biswas^2^ | 2018 | Bangladesh | Retrospective case series | 450 case files reviewed of women who received MCoC.  72 of these women were referred to hospital.  In-depth review of 15 randomly selected files. | Five tea gardens in Sylhet division, Moulvibazar District, Bangladesh  (an area with high maternal and perinatal mortality) | Midwifery led continuity of care service implemented.  Midwives provide antenatal, intrapartum and postnatal clear, however no evidence of one-to-one allocation or 'lead/known midwife' | Novel |
| Dery^3^ | 2020 | Ghana | Retrospective review of continuity of care received. | 14,474 women claiming National Health Insurance for antenatal and birth care. | Volta region (rural) | Study sought to evaluate level of continuity of care received by women in the region using National Health insurance data.  Continuity assessed as high but by service, not clinician or team provider. | Evaluation of program |
| Gu^4^ | 2013 | China | Randomised controlled trial. | 110 women enrolled and allocated to either midwife-led or obstetrician-led antenatal care. | Antenatal midwifery clinic in a large urban hospital in Shanghai. | Midwife led clinic within a large hospital.  Midwives on-call to attend to women in labour, that they had seen for antenatal care, unless midwife was on sick leave or needed time off for attendance at a previous long labour. | Novel |
| Hailemeskel^5 6^ | 2021, 2021 | Ethiopia | Quasi-experimental design. Women from four primary hospitals allocated to either midwife-led continuity of care or a shared model of care. | 1178 women in total, 589 in the intervention group (midwife-led care) and 589 in the comparison group. | Four selected hospitals from within the North Shoa zone, Amhara regional state.  Maternity care provided via 95 health centres and 389 health posts with one referral hospital providing emergency obstetric care. | Midwife-led continuity of care. Women who received their entire antenatal, labour, birth and postnatal care by one midwife from the midwife-led continuity of care group were included in the intervention group. | Novel |
| Hua^7^ | 2018 | China | Cohort study comparing women in obstetrician-led care and midwife-led care. | 1730 women from 9 hospitals in Shanghai.  1568 participants completed a survey. | Urban centres in Shanghai. | Women could elect to have care through Continuity of Midwifery care teams that operate concurrently with obstetric led teams in selected hospitals.  Midwives provided antenatal, intrapartum and postnatal care. Difficult to discern degree of 'known-provider'. | Novel |
| Liambilia^8^ | 2013, 2014 | Kenya | Pre- and post-evaluation of a 6-month long initiative (February to July 2011) to implement community midwifery services.  The initiative included revision of guidelines, training/updates, provision of equipment, and promotion of community referral systems. | 294 cases and 291 controls. | Low resource setting with high number of women birthing at home with traditional birth attendants. | Community-based midwives offered antenatal, intrapartum and postnatal care, as well as family planning and HIV services.  No known midwife or one-to-one allocation explicitly described however, midwives were based in the local community and around half of women had their first antenatal visit and birth in the midwife’s home. | Scale up of ‘community midwifery approach’.  First piloted by Kenya’s Division of Reproductive Health, Ministry of Health and the Population Council in Western Province in 2005. |
| Liu^9^ | 2021 | China | Cross-sectional survey of 4192 women who had a vaginal birth in a maternity centre in Shanghai between March and June2019. | 4192 women using a self-administered questionnaire. | Shanghai First Maternity and Infant Hospital - one of Chinas largest providers of maternity care. | Hospital employed midwives to provide care for all women wishing to have a vaginal birth. Antenatal, Intrapartum and Postnatal care provided.  Degree of 'known-provider' limited to intrapartum and postpartum care usually by the same midwife. | Part of current policy in China |
| Mortensen^10-12^ | 2018, 2019, 2019 | Palestine | Three studies report on this initiative:  1. Non-randomised Cluster Interventions study (2018).  2. Register-based retrospective cohort study (2019)  3. A case-control study comparing maternal satisfaction between Continuity of Care and standard practice. | 1. 14 clinics in intervention arm and 25 clinics in control arm.  2. 2201 singleton births January 2016 - July 2017.  3. 200 women (100 in intervention arm (Continuity of Care) and 100 in the control arm). | Nablus region in occupied Palestine, involving a government hospital and ten rural villages. West Bank, Palestinian women affected by ongoing Israeli occupation and difficulty accessing care. | Midwives employed at government hospitals deployed to community clinic sites for antenatal care and postnatal home visits i. Each midwife allocated a geographic area including 50-100 pregnant women. | Novel |
| Rosyidah^13^ | 2019 | Indonesia | Mixed methods evaluation of women’s experience of midwifery continuity of care by student midwives | 41 women | Bangetayu Public Health Centre Semarang | Continuity of midwifery care provided by student midwives during antenatal (third trimester), intrapartum and postpartum periods. | Unclear |
| Rahman^14^ | 2021 | Bangladesh | Reports on Midwife Led Care Centre over a 3-year period at the Charikata Union Health and Family Welfare Centre in Jaintiapur, Sylhet, Bangladesh. | 2540 women delivered with the service during the 3-year period. | Midwife led care centre within a rural government facility in Jaintiapur Sylhet, Bangladesh. | A team of four midwives provide antenatal, intrapartum care, visit postnatal women at home and provide family planning services. No evidence of one-to-one midwife allocation, but small team of four midwives and so likely to be known to women. | Novel |
| Shahinfar^15^ | 2021 | Iran | Qualitative exploration of women’s views of midwifery continuity of care (part of a larger mixed-methods study) | 15 low-risk primipara interviewed | Private midwifery clinic and Hospital in Ahvaz city, Iran. | Small midwifery continuity of care team who established private midwifery clinic and booked women for birthing at two local hospitals.  Full continuity of care from 24 weeks gestation to 6 weeks postpartum. | Novel |
| Thommesen^16^ | 2020 | Afghanistan | Explored women’s perceptions of midwifery care. | 14 women interviewed (including 4 midwives). | 4 villages in Kunar and Lagnan rural provinces | Education of locally recruited midwives and provision of maternity clinics within walking distance.  No explicit system of allocating midwives to women, however midwives were recruited from local villages and known to the women. | Novel education initiative |
| Yanti^17^ | 2015 | Indonesia | Education intervention evaluation - intervention testing compared students understanding to a control group of students that had not had not been exposed to continuity of care. | 54 midwifery students understanding of continuity of care was evaluated after exposure to continuity of midwifery during 6 months of clinical training.  52 midwifery students each provided care to 2-3 women across the continuum | Urban and rural | Follow-through style of clinical education of student midwives. Antenatal, Intrapartum and Postnatal Care provided by midwifery students. | Novel education initiative |

**References**

1. Anwar S, Jan R, Qureshi RN, et al. Perinatal women's perceptions about midwifery led model of care in secondary care hospitals in Karachi, Pakistan. *Midwifery* 2014;30(3):e79-90. doi: 10.1016/j.midw.2013.10.020 [published Online First: 2013/12/03]

2. Biswas A, Anderson R, Doraiswamy S, et al. Timely referral saves the lives of mothers and newborns: midwifery led continuum of care in marginalized teagarden communities - a qualitative case study in Bangladesh. *F1000Research* 2018;7:365. doi: 10.12688/f1000research.13605.1

3. Dery S, Aikins M, Maya E. Longitudinal continuity of care during antenatal and delivery in the Volta Region of Ghana. *International Journal of Gynecology & Obstetrics* 2020;151(2):219-24. doi: 10.1002/ijgo.13301

4. Gu CWXDYZXZZ. The effectiveness of a Chinese midwives' antenatal clinic service on childbirth outcomes for primipare: A randomised controlled trial. *International Journal of Nursing Studies* 2013;50:1689-97. doi: 10.1016/j.ijnurstu.2013.05.001

5. Hailemeskel S, Alemu K, Christensson K, et al. Midwife-led continuity of care improved maternal and neonatal health outcomes in north Shoa zone, Amhara regional state, Ethiopia: A quasi-experimental study. *Women and Birth* 2021 doi: 10.1016/j.wombi.2021.08.008 [published Online First: 2021/09/08]

6. Hailemeskel S, Alemu K, Christensson K, et al. Health care providers' perceptions and experiences related to Midwife-led continuity of care-A qualitative study. *PLoS ONE* 2021;16(10):e0258248. doi: https://dx.doi.org/10.1371/journal.pone.0258248

7. Hua J, Zhu L, Du L, et al. Effects of midwife-led maternity services on postpartum wellbeing and clinical outcomes in primiparous women under China's one-child policy. *BMC Pregnancy & Childbirth* 2018;18(1):329. doi: https://dx.doi.org/10.1186/s12884-018-1969-9

8. Liambila W, Obare F, Undie C, et al. The community midwifery model in Kenya: Expanding access to comprehensive reproductive health services at the community level. *African Journal of Midwifery and Women's Health* 2013;7(4):171-77. doi: 10.12968/ajmw.2013.7.4.171

9. Liu Y, Li T, Guo N, et al. Women's experience and satisfaction with midwife-led maternity care: a cross-sectional survey in China. *BMC Pregnancy & Childbirth* 2021;21(1):151. doi: https://dx.doi.org/10.1186/s12884-021-03638-3

10. Mortensen B, Diep LM, Lukasse M, et al. Women's satisfaction with midwife-led continuity of care: an observational study in Palestine. *BMJ Open* 2019;9(11):e030324. doi: https://dx.doi.org/10.1136/bmjopen-2019-030324

11. Mortensen B, Lieng M, Diep LM, et al. Improving maternal and neonatal health by a midwife-led continuity model of care - an observational study in one governmental hospital in Palestine. *EClinicalMedicine* 2019;10:84-91. doi: 10.1016/j.eclinm.2019.04.003 [published Online First: 2019/06/14]

12. Mortensen B, Lukasse M, Diep LM, et al. Can a midwife-led continuity model improve maternal services in a low-resource setting? A non-randomised cluster intervention study in Palestine. *BMJ Open* 2018;8(3):e019568. doi: https://dx.doi.org/10.1136/bmjopen-2017-019568

13. Rosyidah H, Aisyaroh N. Clients’ satisfaction with continuity of midwifery care. *Jurnal Kesehatan Ibu dan Anak* 2018;12(2):129~35. doi: 10.29238/kia.v12i2.149

14. Rahman S. Midwife led Care Centre in a Government Facility: Charikata Union Health and Family Welfare Centre (UH&FWC), Jaintiapur, Sylhet. 2021 Wednesday, Apr 20, 2022.

15. Shahinfar S, Abedi P, Najafian M, et al. Women's perception of continuity of team midwifery care in Iran: a qualitative content analysis. *BMC Pregnancy Childbirth* 2021;21(1):173. doi: 10.1186/s12884-021-03666-z [published Online First: 2021/03/04]

16. Thommesen T, Kismul H, Kaplan I, et al. "The midwife helped me ... otherwise I could have died": women's experience of professional midwifery services in rural Afghanistan - a qualitative study in the provinces Kunar and Laghman. *BMC Pregnancy & Childbirth* 2020;20(1):140. doi: https://dx.doi.org/10.1186/s12884-020-2818-1

17. Yanti Y, Claramita M, Emilia O, et al. Students' understanding of "Women-Centred Care Philosophy" in midwifery care through Continuity of Care (CoC) learning model: a quasi-experimental study. *BMC Nursing* 2015;14:22-22. doi: 10.1186/s12912-015-0072-z
