# Supplementary material for: Midwifery continuity of care: A scoping review of where, how, by whom and for whom?
Source: PLOS Glob Public Health. 2022 Oct 5;2(10):e0000935. doi: 10.1371/journal.pgph.0000935 (PMC10021789; doi:10.1371/journal.pgph.0000935)
Supplement: S3 Table — (DOCX) [file pgph.0000935.s004.docx]

**S3 Table: Major model categories in MaCCS**^(1)^

| **Major Model Category** | **Description** |
| --- | --- |
| 1. Private obstetrician (specialist) care | Antenatal care provided by a private specialist obstetrician. Intrapartum care is provided in either a private or public hospital by the private specialist obstetrician and hospital midwives in collaboration. Postnatal care is usually provided in the hospital by the private specialist obstetrician and hospital midwives and may continue in the home, hotel or hostel. |
| 1. Private midwifery care | Antenatal, intrapartum and postnatal care is provided by a private midwife or group of midwives in collaboration with doctors in the event of identified risk factors. Antenatal, intrapartum and postnatal care could be provided in a range of locations including the home. |
| 1. General Practitioner obstetrician care | Antenatal care provided by a GP obstetrician. Intrapartum care is provided in either a private or public hospital by the GP obstetrician and hospital midwives in collaboration. Postnatal care is usually provided in the hospital by the GP obstetrician and hospital midwives and may continue in the home or community. |
| 1. Shared care | Antenatal care is provided by a community maternity service provider (doctor and/or midwife) in collaboration with hospital medical and/or midwifery staff under an established agreement, and can occur both in the community and in hospital outpatient clinics. Intrapartum and early postnatal care usually takes place in the hospital by hospital midwives and doctors, often in conjunction with the community doctor or midwife (particularly in rural settings). |
| 1. Combined care | Antenatal care provided by a private maternity service provider (doctor and/or midwife) in the community. Intrapartum and early postnatal care provided in the public hospital by hospital midwives and doctors. Postnatal care may continue in the home or community by hospital midwives. |
| 1. Public hospital maternity care | Antenatal care is provided in hospital outpatient clinics (either onsite or outreach) by midwives and/or doctors. Care could also be provided by a multidisciplinary team. Intrapartum and postnatal care is provided in the hospital by midwives and doctors in collaboration. Postnatal care may continue in the home or community by hospital midwives. |
| 1. Public hospital high risk maternity care | Antenatal care is provided to women with medical high risk/complex pregnancies by maternity care providers (specialist obstetricians and/or maternal-fetal medicine subspecialists in collaboration with midwives) with an interest in high risk maternity care in a public hospital. Intrapartum and postnatal care is provided by hospital doctors and midwives. Postnatal care may continue in the home or community by hospital midwives. |
| 1. Team midwifery care | Antenatal, intrapartum and postnatal care is provided by a small team of rostered midwives (no more than eight) in collaboration with doctors in the event of identified risk factors. Intrapartum care is usually provided in a hospital or birth centre. Postnatal care may continue in the home or community by the team midwives. |
| 1. Midwifery Group Practice caseload care | Antenatal, intrapartum and postnatal care is provided within a publicly-funded caseload model by a known primary midwife with secondary backup midwife/midwives providing cover and assistance with collaboration with doctors in the event of identified risk factors. Antenatal care and postnatal care is usually provided in the hospital, community or home with intrapartum care in a hospital, birth centre or home. |
| 1. Remote area maternity care | Antenatal and postnatal care is provided in remote communities by a remote area midwife (or a remote area nurse) or group of midwives sometimes in collaboration with a remote area nurse and/or doctor. Antenatal care may also be provided via telehealth or fly-in-fly-out clinicians in an outreach setting. Intrapartum and early postnatal care is provided in a regional or metropolitan hospital (involving temporary relocation prior to labour) by hospital midwives and doctors. |
| 1. Private obstetrician and privately practising midwife joint care | Antenatal, intrapartum and postnatal care is provided by a privately practising obstetrician and midwife from the same collaborative private practice. Intrapartum care is usually provided in either a private or public hospital by the privately practising midwife and/or private specialist obstetrician in collaboration with hospital midwifery staff. Postnatal care is usually provided in the hospital and may continue on in the home, hotel or hostel by the privately practising midwife |

**Source**

1. Donnolley N, Butler-Henderson K, Chapman M, Sullivan E. The development of a classification system for maternity models of care. Health Information Management Journal. 2016;45(2):64-70.
