# Supplementary material for: Midwifery continuity of care: A scoping review of where, how, by whom and for whom?
Source: PLOS Glob Public Health. 2022 Oct 5;2(10):e0000935. doi: 10.1371/journal.pgph.0000935 (PMC10021789; doi:10.1371/journal.pgph.0000935)
Supplement: Attachment — Submitted filename: Response to Review Midwifery Continuity of Care R2.docx [file pgph.0000935.s006.docx]

**PGPH-D-22-00905
Midwifery continuity of care: A scoping review of where, how, by whom and for whom?
PLOS Global Public Health**

Dear Ahmed Waqas

We contacted ESRI for consent to publish these images in PLOS, under the CC BY 4.0 license. Using your advice. Unfortunately we have had no response in over a week. Hence we have removed the figure and any reference to it.

Thank you
